# Quantification of Bioactive Compounds by HPLC-ESI-MS/MS and Evaluation of Antioxidant and Enzyme Inhibitory Activities of Acorn Flour Extracts

**DOI:** 10.3390/antiox13121526

**Published:** 2024-12-13

**Authors:** Laura Acquaticci, Agnese Santanatoglia, Elena Vittadini, Daniela Beghelli, Antonietta La Terza, Gokhan Zengin, Giovanni Caprioli

**Affiliations:** 1 Chemistry Interdisciplinary Project (ChIP), School of Pharmacy, University of Camerino, Via Madonna delle Carceri 9/B, 62032 Camerino, Italy; laura.acquaticci@unicam.it (L.A.); agnese.santanatoglia@unicam.it (A.S.); 2 School of Biosciences and Veterinary Medicine, University of Camerino, Via Gentile III da Varano, 62032 Camerino, Italy; elena.vittadini@unicam.it (E.V.); daniela.beghelli@unicam.it (D.B.); antonietta.laterza@unicam.it (A.L.T.); 3 Physiology and Biochemistry Laboratory, Department of Biology, Science Faculty, Selcuk University, Konya 42130, Turkey; gokhanzengin@selcuk.edu.tr

**Keywords:** acorn flour, polyphenols, *Quercus robur* (Fagaceae), antioxidant activity, enzyme inhibition, bioactive compounds, nutraceuticals

## Abstract

This study provides the first comprehensive evaluation of the bioactive potential of acorn flour extracts (*Quercus robur*, Fagaceae) prepared at different temperatures (20, 60, 80 and 100 °C), focusing on polyphenolic content, antioxidant properties and enzyme inhibitory activities. Through HPLC-ESI-MS/MS analysis, 36 bioactive compounds were identified, with the extract at 60 °C showing the highest concentrations of key polyphenols, notably gallic acid (210,008.9 mg·kg^−1^) and ellagic acid (45,469.6 mg·kg^−1^). This extract also exhibited a high antioxidant activity and significant inhibition of glucosidase and acetylcholinesterase, suggesting potential benefits for diabetes management and neuroprotection. The results indicate that extraction temperature affects bioactivity, with the 60 °C extract standing out as a promising candidate for nutraceutical, pharmaceutical, and cosmeceutical applications due to its rich polyphenol profile and potent biological properties.

## 1. Introduction

Acorns, the nuts of the *Quercus genus* (Fagaceae), have been long valued across various cultures, especially in the Mediterranean area, for their nutritional richness and versatility in culinary uses, due to their high content of carbohydrates, proteins, and essential minerals, including calcium, iron, potassium, phosphorus, magnesium, and vitamins A and C [1,2,3]. Traditionally included in human diets, acorns have been associated with numerous health benefits, with several studies indicating that *Quercus* extracts may contribute to cancer prevention [4,5]. Historically, acorns have been used to produce flour, bread, and jelly; particularly in regions where oak trees are prevalent, such as North America, Europe and parts of Asia [6]. Giving the increasing interest in natural antioxidants and enzyme inhibitors for the prevention of chronic conditions like diabetes and neurodegenerative diseases, there is a need to investigate underutilized plant sources like acorn flour. Despite their nutritional benefits, acorn utilization in the food fields remains limited, primarily due to the presence of tannins, which exhibit antinutritive effects, such as inhibiting protein digestion and imparting a bitter taste [7,8]. Recent studies, however, have underscored the potential of acorns in contemporary food applications, attributed to their polyphenols, including gallic acid, ellagic acid and tocopherols, which possess strong antioxidant, antibacterial, and antitumor properties [9,10]. Moreover, these bioactive compounds in acorns have been found to possess anti-inflammatory, anti-adipogenic and neuroprotective activities, which contribute positively to health and wellness [11,12,13,14]. Additionally, acorn extracts have also shown antimicrobial effects, mostly in inhibiting biofilm formation [15]. Other research has revealed that acorns contain significant levels of unsaturated fatty acids, and they are a potent source of dietary fiber, both critical for preventing chronic diseases such as obesity and cardiovascular issues [16]. The unique phytochemical composition of acorns, rich in antioxidants and beneficial compounds, indicates their promising potential for use in functional foods, pharmaceuticals, and cosmetic products [12,13]. Nowadays, global demand for sustainable, health-enhancing food sources has brought renewed attention to acorns, especially in major producing countries like China, where they are expected as an important resource to stimulate economic development and alleviate poverty in rural communities [17]. Recently, acorn flour has also gained interest for its potential in gluten-free baking [18]: its high fiber content and gluten-free nature make it an attractive alternative, for improving the nutritional quality and sensory properties of bakery products. Recent studies have demonstrated the feasibility of incorporating acorn flour into bread, cakes, and biscuits, also highlighting its ability to enhance the nutritional value of these products [18,19,20]. In this research, for the first time, extracts from *Q. robur* were prepared at different temperatures, and their polyphenolic content, antioxidant activity and enzyme inhibitory potential were evaluated. This analysis aimed to further understand their potential applications in nutraceutical, pharmaceutical and cosmeceutical industries.

## 2. Materials and Methods 

### 2.1. Standards and Reagents

The standards cyanidin 3-glucoside chloride, delphinidin 3,5-diglucoside chloride, delphinidin 3-galactoside chloride, petunidin 3-glucoside chloride, malvidin 3-galactoside chloride, quercetin 3-glucoside and kaempferol 3-glucoside were procured from PhytoLab (Vestenbergsgreuth, Germany). The remaining 29 of the 36 total phenolic compound standards were obtained from Sigma-Aldrich (Milan, Italy). Quantification of polyphenols has been carried out with authentic analytical standard for all compounds. Formic acid (99%) was sourced from Merck (Darmstadt, Germany) and hydrochloric acid (37%) from Carlo Erba Reagents (Milan, Italy). HPLC-grade methanol was supplied by Sigma-Aldrich (Milano, Italy). Deionized water was purified using a Milli-Q SP Reagent Water System (Millipore, Bedford, MA, USA). All solvents and solutions were filtered through a 0.2 μm polyamide filter from Sartorius Stedim (Goettingen, Germany). For biological activity assays, chemicals were obtained from Sigma-Aldrich (Darmstadt, Germany). All reagents were of analytical grade.

### 2.2. Acorn Sample

The acorn flour used in this study was commercially purchased from Dary Natury (Koryciny, Poland; https://darynatury.pl/ accessed on 5 February 2024) and it is composed of dried and ground organic acorns of the pedunculate oak (*Q. robur*), as declared in the product’s technical specifications. The declared nutritional composition in the label is as follows: energy value 1355 kJ/322 kcal; fat 5.1 g; including saturated fatty acids 0.9 g; carbohydrates 73.4 g; including sugars 9.9 g; fiber 16.8 g; protein 4.1 g; salt 0.0 g; potassium 450 mg; calcium 74 mg; phosphorus 73 mg; magnesium 34 mg; vitamin B3 2.1 mg.

### 2.3. Extract Preparation

**Figure 1:**
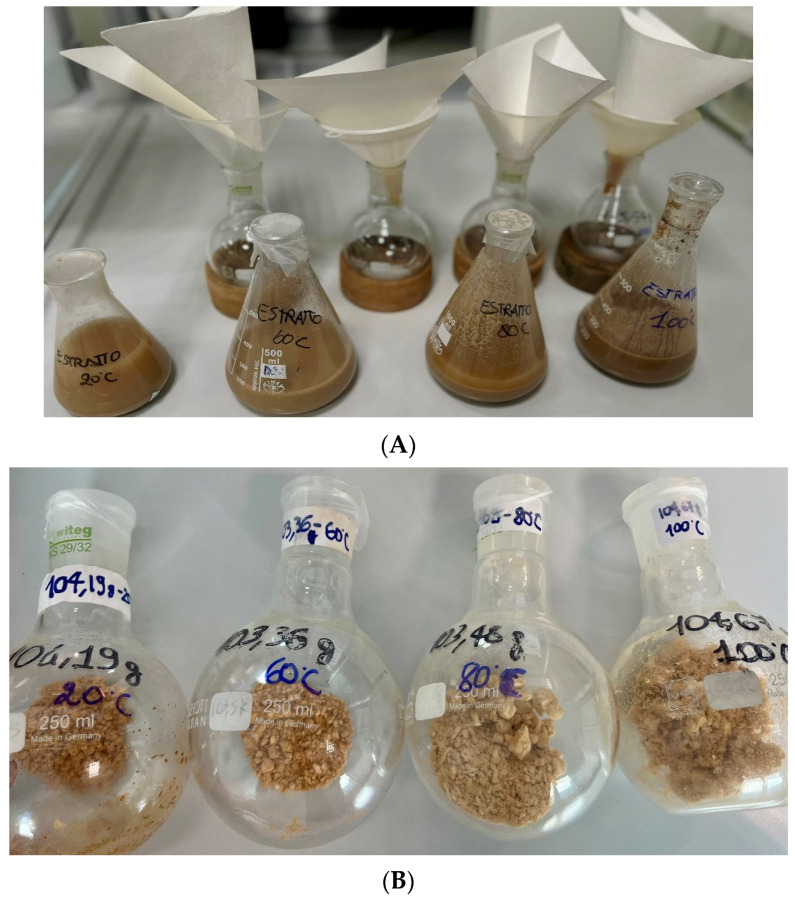
Preparation and visual appearance of acorn flour extracts at different temperatures. (**A**) The extraction process involves mixing acorn flour with water and maintaining a 1:10 (flour) ratio, followed by extraction at 20 °C, 60 °C, 80 °C, and 100 °C under magnetic stirring for 30 min. (**B**) The resultant liquid extracts were freeze-dried to obtain lyophilized extracts, which were stored at −20 °C in the dark until further analysis.

For the extraction, 20 g of acorn flour was mixed with 200 mL of water, maintaining a 1:10 (flour/water) ratio and then extracted at 20 °C, 60 °C, 80 °C, and 100 °C under magnetic stirring for 30 min. Once the extraction was complete, the solution was filtered through filter paper to separate any solid materials and the resultant liquid extract was collected (Figure 1). This extract was then freeze-dried and stored at −20 °C in the dark until further analysis. The extraction yields of the four extracts were, respectively, of 4.7%, 4.5%, 3.9% and 3.7% for 60 °C, 20 °C, 80 °C, and 100 °C, expressed as *w*/*w* dry weight.

### 2.4. Polyphenolic Content Though High-Performance Liquid Chromatography–Electrospray Ionization–Mass Spectrometry (HPLC–ESI–MS/MS) Analysis

For HPLC analyses, 5 mg of the lyophilized extract was dissolved in 5 mL of methanol (1 mg·mL^−1^). The solution was sonicated for 10 min to ensure complete dissolution, then centrifuged and filtered. Methanol was selected as dissolving solvent to prepare the sample for HPLC-MS/MS analysis because it is used also in the mobile phase in the chromatographic run. HPLC-ESI-MS/MS setup consisted of an Agilent 1290 Infinity series coupled to a Triple Quadrupole 6420 mass spectrometer (Agilent Technologies, Santa Clara, CA, USA), using an electrospray ionization source in both positive and negative modes. For compound separation, a Synergi Polar-RP C18 column (250 mm × 4.6 mm, 4 μm) from Phenomenex (Cheshire, UK) was employed, with a guard cartridge in place. The mobile phase utilized a gradient flow of water (A) and methanol (B), both containing 0.1% formic acid, at 0.8 mL/min. Gradient steps included an initial 20% B held isocratically, rising to 85% B within 25 min, then returning to 20% B for requilibration. Injection volume was 2 μL, with a column temperature of 30 °C. Ion source conditions were 350 °C drying gas, 12 L/min flow, 55 psi nebulizer and 4000 V capillary voltage. Detection operated in dynamic-MRM mode, based on previously published method [21,22,23] (Table S1). Validation parameters (equation of the calibration curve, linearity, LOQ and LOD) were reported in Table S2.

### 2.5. Polyphenolic Content Through TPC and TFC

The general polyphenolic content was assessed using two in vitro methods previously developed [24]. Total phenolic content (TPC) and total flavonoid content (TFC) were measured using procedures developed previously [25]. Briefly, TPC was determined using the Folin–Ciocalteu method, in which 0.5 mL of methanolic solution of the extract (5 mg of lyophilized extract in 5 mL of MeOH) was added with 2.5 mL of Folin–Ciocalteu reagent and 7 mL of 7.5% aqueous solution of Na_2_CO_3_ and left in the dark for 2 h. TFC was determined by adding 150 μL NaNO_2_ (0.5 M), 3.2 mL methanol (30% *v*/*v*) and 150 μL AlCl_3_ (0.3 M) to 500 μL of methanolic solution of the extract (5 mg of lyophilized extract in 5 mL of MeOH). The values of TPC and TFC were measured using Agilent Cary 8454 UV–Vis spectrophotometer (Santa Clara, CA, USA) at 765 nm and 506 nm, respectively. Results were expressed as mg of gallic acid equivalents per g of dry extract (mg GAE/g DE) and as mg rutin equivalents per g of dry extract (mg RE/g DE), respectively.

### 2.6. Antioxidant Test

The antioxidant activity was assessed by using six tests (DPPH, ABTS, CUPRAC, FRAP, MCA and PBD). Antioxidant activities for DPPH, ABTS, CUPRAC and FRAP assays were recorded as mg of trolox equivalents (TE) per gram of extract. Metal chelating activity (MCA) was reported in mg of disodium EDTA equivalents (EDTAE) per gram and the phosphomolybdenum (PBD) assay results were expressed as mmol of trolox equivalents (TE) per gram [24].

### 2.7. Enzyme Inhibitory Test

Enzyme inhibition assays followed previous published protocols from [24]. Inhibitory activity for amylase and glucosidase was reported as mmol of acarbose equivalents (ACAE) per gram of extract, while acetylcholinesterase (AChE) and butyrylcholinesterase (BChE) inhibition was expressed in mg of galanthamine equivalents (GALAE) per gram. Tyrosinase inhibition was measured as mg of kojic acid equivalents (KAE) per gram of extract [24].

### 2.8. Statistical Analysis

Statistical analysis was performed using XLSTAT (Version 16). All tests were conducted in triplicate (n = 3) and results are expressed as mean ± standard deviation (SD), with relative standard deviations (RSD %) kept below 20%. Differences among samples were assessed using one-way ANOVA, with a significance threshold of *p* < 0.05. Pearson correlation analysis was conducted using MetaboAnalyst 5.0 tool (https://www.metaboanalyst.ca/ accessed on 8 April 2024) to explore the relationships between polyphenols and spectrophotometric assays, as well as between polyphenols and enzyme inhibition assays. The Pearson r algorithm was involved to measure the linear relationship between the different variables and to assess the strength and direction of the association between them.

## 3. Results and Discussions

### 3.1. Bioactive Compounds

A total of 36 bioactive compounds were quantified in acorn flour extracts using HPLC-ESI-MS/MS, as presented in Table 1 and Figure S1. The acorn flour extract at 60 °C exhibited the highest concentration of phenolic compounds, reaching 262,665.54 mg·kg^−1^. The primary phenolics contributing to this result were gallic acid (210,008.9 mg·kg^−1^), rutin (38.24 mg·kg^−1^), ellagic acid (45,469.64 mg·kg^−1^), quercetin (26.47 mg·kg^−1^), kaempferol (1597.79 mg·kg^−1^), and isorhamnetin (126.67 mg·kg^−1^), which were significantly higher than in other samples, even if with no with statistically significant differences. Regarding acorn extract at 20 °C, the levels of procyanidin B2 (126.25 mg·kg^−1^), epicatechin (44.69 mg·kg^−1^), vanillic acid (553.21 mg·kg^−1^), pelargonidin-3-rutinoside (3.84 mg·kg^−1^), ferulic acid (45.29 mg·kg^−1^), 3,5-dicaffeoylquinic acid (63.90 mg·kg^−1^) and phloretin (2.54 mg·kg^−1^) were higher compared to other samples. Finally, delphinidin-3-galactoside (11.32 mg·kg^−1^), delphinidin-3,5-diglucoside (850.65 mg·kg^−1^) and kaempferol-3-glucoside (57.65 mg·kg^−1^) were most efficiently extracted at 80 °C. In general, the increment of temperature (from 20 °C to 60–80 °C) seemed to be related to an increment of concentrations of anthocyanins. The lowest concentrations of phenolic compounds were found in acorn flour extracts at 100 °C, except for catechin (842.63 mg·kg^−1^), malvidin-3-galactoside (2.31 mg·kg^−1^) and hyperoside (1090.40 mg·kg^−1^). These results confirm the influence of temperature on the extraction efficiency and stability of polyphenols. In fact, ref. [26] reported that the best extraction yields of polyphenols in pomegranate peels using ultrasound were obtained with a temperature of 50–60 °C. The polyphenolic content and antioxidant activities of acorn flour are well-documented [11,12,13,14,15]. Specifically, acorn extracts from *Quercus canariensis* (Fagaceae) have been reported to possess a phenolic profile comprising 19 compounds, with coumarin being the most abundant, alongside gallic, syringic and trans-ferulic acids and kaempferol as the major flavonoid compound [27]. Additionally, ref. [28] reported that acorn extracts from different Quercus species show high phenolic content, with chlorogenic acid and coumarin as dominant compounds. Furthermore, acorn oil has been identified as a rich source of phenolic compounds, including polyphenols and tocopherols, with γ-tocopherol being the most abundant [29]. Comparing the content of polyphenols in acorn flour with that in wheat flour, the concentration of ferulic acid was lower in acorn flour, while the concentration of p-hydroxybenzoic, vanillic, syringic, and p-coumaric acids were higher in acorn flour [30,31].

### 3.2. Antioxidant Activity (TPC and TFC)

Polyphenolic content was determined also using TPC and TFC assays. The total phenolic content (TPC) and total flavonoid content (TFC) (Table 2 and Figure S2) served as indicators of phenolic concentration in the extracts, due to their association with antioxidant properties [32,33]. No significant variation in TPC was observed with temperature (no statistically significant differences among the samples), with values ranging from 103.21 mg GAE/g at 100 °C to 105.39 mg GAE/g at 80 °C. The relationship between temperature and phenolic content is complex, as TPC decreased from 40 °C to 60 °C, then increased to 80 °C, followed by a reduction to 100 °C. The highest TFC value was noted in the 60 °C extract (6.28 mg RE/g), showing statistically significant differences compared to other extracts. Especially, the lowest TFC value was observed at 100 °C, suggesting that elevated temperatures may reduce flavonoid concentration in the extracts.

### 3.3. Antioxidant Activity (DPPH, ABTS, CUPRAC, FRAP, MCA and PBD)

In both food and biological systems, the generation of free radicals is directly linked with oxidation processes [29] and their presence in biological systems can contribute to the development of various diseases [30]. Several studies have investigated the antioxidant activity in different acorn species and related products [18,20,34,35,36,37,38]. However, specific information about *Q. robur* remains limited [34]. To evaluate the antioxidant activity of acorn flour extracts, six different assays were conducted (DPPH, ABTS, CUPRAC, FRAP, MCA and PBD), with results reported in Table 3 and Figure S3. The antioxidant activity was assessed through the 2,2-diphenyl-1-picrylhydrazyl (DPPH) radical scavenging assay (Figure S3B), where the extract at 60 °C showed the highest antioxidant activity (493.57 mg TE/g), although without statistically significant differences with other extracts. This aligns with the TFC findings, indicating that optimal flavonoid extraction at this temperature enhances antioxidant activity. Consistently, the lowest DPPH value was observed at 100 °C. In the ABTS assay, which involves reduction in the ABTS·+ (2,2′-azino-bis-3-ethylbenzthiazoline-6-sulphonic acid) radical by antioxidants, the highest value was recorded in the 60 °C extract (964.53 mg TE/g), showing statistically significant differences compared to other values, while the lowest value was observed at 100 °C (796.85 ± 4.33 mg TE/g). Previous studies have also shown the antioxidant capacity of acorn extracts, as water and methanol extracts from acorn exhibit scavenging activities against DPPH and ABTS radicals, suggesting their potential as antioxidants [39]. The CUPRAC assay (cupric reducing antioxidant capacity) revealed the highest result for the 60 °C extract (951.57 mg TE/g) with statistical differences, while the lowest was at 100 °C (700.17 mg TE/g). Similarly, the FRAP assay, which measures ferric reducing ability, confirmed higher activity with 60 °C extract (730.48 mg TE/g) compared to the lowest activity at 100 °C (562.16 ± 3.78 mg TE/g), consistent with previous findings. The metal chelating assay (MCA) demonstrated the highest ion-binding capacity at 80 °C (26.97 mg EDTAE/g), with statistically significant differences from other samples. Lastly, the phosphomolybdenum assay (PBD) indicated the highest antioxidant activity for the 60 °C extract (5.10 mmol TE/g), which was statistically distinct from other samples. In summary, the acorn flour extract at 60 °C exhibited high phenolic content (TPC and TFC) and important antioxidant activity across most assays, except for MCA, which peaked at 80 °C. These results highlight the potential of acorn flour extracts, particularly the 60 °C extract, as natural sources of antioxidants.

### 3.4. Enzyme Inhibitory Activity

The enzyme inhibitory activity was assessed through various assays targeting cholinesterase (AChE), butyrylcholinesterase (BChE), tyrosinase, amylase and glucosidase inhibition. The results are provided in Table 4 and Figure S4. Alzheimer’s disease (AD), a neurodegenerative disorder, manifests with symptoms such as cognitive decline, psychosis, hyperactivity, aggression, and depression. Inhibitors of AChE reduce its synaptic activity, but often cause side effects like nausea, vomiting, anorexia, diarrhea, and bradycardia [40]. Safer alternatives from natural sources are increasingly sought for AD prevention and treatment, prompting significant interest in plant-derived anti-AD compounds. The extract at 60 °C exhibited the highest AChE inhibitory activity (2.09 mg GALAE/g), showing a statistically significant difference from the extracts at 80 °C and 100 °C (*p* < 0.05). However, BChE inhibition did not show statistically significant differences across samples, with values ranging between 2.21 and 2.57 mg GALAE/g. Tyrosinase catalyzes the melanin synthesis pathway, affecting skin pigmentation [41]. Plants are a primary source of tyrosinase inhibitors, which are considered non-toxic and have potential for skin-lightening applications. Tyrosinase inhibition can be beneficial for treating hyperpigmentation disorders like age spots, freckles and melasma. Also in this case, the extract at 60 °C showed the highest inhibition of tyrosinase (22.66 mg KAE/g), which was statistically different from the extracts at 20 °C, 80 °C and 100 °C. Diabetes mellitus (DM), a common endocrine disorder, involves disturbances in carbohydrate, lipid and lipoprotein metabolism, leading to hyperglycemia and complications such as hyperlipidemia, hyperinsulinemia, hypertension and atherosclerosis [42]. α-Amylase and α-glucosidase are key enzymes in carbohydrate digestion and their inhibition can help control blood glucose levels, providing a potential preventive approach for hyperglycemia [43,44]. The extract showed higher α-amylase inhibition at 20 °C, 60 °C and 100 °C, with values between 0.051 and 0.056 mmol ACAE/g. For α-glucosidase, the 60 °C extract displayed the highest inhibition (0.986 mmol ACAE/g), statistically distinct from the 20 °C and 100 °C extracts. These findings highlight the potential of α-glucosidase inhibitors in acorn flour extracts for managing diabetes. The role of α-amylase and α-glucosidase inhibition in developing antidiabetic agents that control postprandial hyperglycemia is well-recognized [45]. Ellagic acid, a component in acorn extracts, has shown important inhibitory effects on α-amylase and α-glucosidase [46]. Additionally, recent research has found that syringic and vanillic acids are effective against α-amylase, while caffeic acid inhibits α-glucosidase [47]. Furthermore, ref. [48] reported that the enzyme-inhibitory effects of acorn extracts may help reduce postprandial blood glucose levels, making them beneficial for diabetes management. In summary, these findings demonstrate that acorn flour extracts possess significant enzyme inhibitory activity, with temperature playing a critical role in enhancing this activity.

### 3.5. Statistical Analysis

#### 3.5.1. Correlation Between Polyphenols and Antioxidant Assays

The Pearson correlation analysis between polyphenolic content and antioxidant assays (DPPH, ABTS, CUPRAC, FRAP, MCA and PBD) demonstrated significant relationships, with corresponding *p*-values confirming the strength of these correlations (*p* < 0.05) (Figure S5 and Tables S3 and S5). Total flavonoid content (TFC) exhibited strong positive correlations with DPPH (r = 0.89, *p* = 0.002) and ABTS (r = 0.92, *p* = 0.001), indicating that flavonoids are major contributors to the free radical scavenging capacity of the acorn flour extracts. Gallic acid, the most abundant phenolic compound at 60 °C, showed a significant positive correlation with both DPPH (r = 0.92, *p* < 0.001) and FRAP (r = 0.85, *p* = 0.005), suggesting its substantial role in antioxidant reduction reactions. Similarly, ellagic acid correlated strongly with ABTS (r = 0.87, *p* = 0.003) and CUPRAC (r = 0.83, *p* = 0.007), reinforcing its importance in overall antioxidant activity. The MCA assay, which measures the metal chelating activity, showed the highest correlation with kaempferol (r = 0.78, *p* = 0.01), suggesting that this flavonoid may contribute to the chelation of metal ions and reduction in oxidative stress. The significant *p*-values (all < 0.05) validate these correlations and indicate that the phenolic compounds, particularly gallic and ellagic acids, play key roles in the antioxidant activity of the acorn extracts. The data suggest that extraction at 60 °C optimizes both the polyphenolic yield and antioxidant potential, supporting the use of these extracts in nutraceutical formulations.

#### 3.5.2. Correlation Between Polyphenols and Enzyme Inhibition Assays

The Pearson correlation analysis between polyphenolic content and enzyme inhibition assays (AChE, BChE, tyrosinase, amylase and glucosidase) provided significant insights into the relationships between individual polyphenols and enzyme inhibitory activities (Figure S6 and Tables S4 and S6). These correlations were validated with *p*-values to confirm their statistical significance (*p* < 0.05). Ellagic acid, which was abundant in the extracts at 60 °C, exhibited a strong positive correlation with glucosidase inhibition (r = 0.88, *p* = 0.003), indicating its significant role in inhibiting this enzyme. This aligns with previous findings that ellagic acid has potential antidiabetic properties due to its capacity to inhibit glucosidase activity. Quercetin and isorhamnetin, both present in significant concentrations, showed strong correlations with AChE inhibition (r = 0.76, *p* = 0.012 for quercetin; r = 0.72, *p* = 0.018 for isorhamnetin), suggesting their potential neuroprotective effects by inhibiting acetylcholinesterase, which is involved in the progression of neurodegenerative diseases such as Alzheimer’s. Kaempferol, another key flavonoid in the extracts, was highly correlated with tyrosinase inhibition (r = 0.85, *p* = 0.005). This finding suggests kaempferol’s potential use in skin depigmentation products due to its inhibitory effect on tyrosinase, the enzyme responsible for melanin production. Catechin displayed a significant positive correlation with amylase inhibition (r = 0.79, *p* = 0.01), suggesting its role in regulating blood glucose levels through the inhibition of amylase, which is crucial for carbohydrate digestion. The strong correlations between specific polyphenols and enzyme inhibition activities highlight the potential therapeutic applications of these compounds, particularly in managing conditions such as diabetes and neurodegenerative diseases. These results underscore the efficacy of acorn flour extracts, particularly at 60 °C, as promising natural sources of enzyme inhibitors.

## 4. Conclusions

In this study, acorn flour extracts of *Quercus robur* species were analyzed, for the first time, by chemical characterization through an HPLC-ESI-MS/MS method. The results highlighted the influence of extraction temperature on the yield of bioactive compounds, with the 60 °C extract showing the highest concentrations of key phenolics such as gallic acid and ellagic acid. The antioxidant assays demonstrated that extracts, particularly at 60 °C, displayed substantial free radical scavenging capacity, correlating strongly with polyphenolic content, especially flavonoids. The enzyme inhibition assays further underscored the therapeutic potential of these extracts, with significant inhibitory effects on enzymes related to neurodegenerative diseases (AChE, BChE), diabetes (amylase, glucosidase) and skin pigmentation (tyrosinase). Correlation analysis revealed that polyphenols like quercetin and isorhamnetin strongly contributed to AChE inhibition, while ellagic acid showed a strong correlation with glucosidase inhibition, supporting its potential antidiabetic properties. The correlations analysis provided clearer insights into the significant relationships between specific polyphenols and both antioxidant and enzyme inhibitory activities, confirming the bioactive potential of the acorn flour extracts, particularly those obtained at 60 °C. These findings suggest that acorn flour extracts hold promise for development in nutraceutical, pharmaceutical and cosmeceutical applications due to their high polyphenol content and potent biological activities.

## Figures and Tables

**Table 1:** Concentrations (mg·kg^−1^) of 38 bioactive compounds in acorn flour extracts at four different temperatures.

No.	Compound	20 °C	60 °C	80 °C	100 °C
1	Gallic acid	155,427.60 ± 26,109.2 *^ab^	210,008.9 ± 23,154.4 ^b^	124,990.6 ± 8609.1 ^a^	117,275 ± 19,841.0 ^a^
2	Neochlorogenic acid	33.94 ± 6.8 ^a^	36.81 ± 2.7 ^a^	34.13 ± 1.6 ^a^	30.87 ± 5.1 ^a^
3	Delphindin-3-galactoside	n.d.	7.71 ± 1.5 ^a^	11.32 ± 1.7 ^a^	9.77 ± 0.0 ^a^
4	Catechin	706.69 ± 17.5 ^a^	821.82 ± 11.3 ^a^	767.36 ± 154.7 ^a^	842.63 ± 168.4 ^a^
5	Procyanidin B2	126.25 ± 0.0 ^a^	n.d.	n.d.	117.93 ± 0.0 ^b^
6	Chlorogenic acid	229.65 ± 5.9 ^a^	246.93 ± 8.5 ^a^	218.39 ± 8.9 ^a^	206.36 ± 40.5 ^a^
7	4-Hydroxy benzoic acid	1253.55 ± 228.1 ^a^	1341.29 ± 135.7 ^a^	1086.54 ± 96.9 ^a^	975.87 ± 192.8 ^a^
8	Epicatechin	44.69 ± 2.2 ^a^	29.88 ± 5.5 ^a^	40.80 ± 3.3 ^a^	42.36 ± 6.2 ^a^
9	Cyanidin-3-glucoside	42.59 ± 4.1 ^a^	57.65 ± 11.2 ^a^	46.39 ± 8.8 ^a^	35.71 ± 6.9 ^a^
10	Petunidin-3-glucoside	n.d.	n.d.	n.d.	n.d.
11	3-Hydroxybenzoic acid	n.d.	n.d.	n.d.	n.d.
12	Caffeic acid	115.99 ± 0.0 ^a^	128.39 ± 1.6 ^a^	119.68 ± 23.8 ^a^	87.91 ± 17.1 ^a^
13	Vanillic acid	553.21 ± 18.9 ^a^	465.02 ± 83.1 ^a^	398.20 ± 79.4 ^a^	403.55 ± 71.8 ^a^
14	Pelargonidin-3-glucoside	21.71 ± 0.0 ^a^	24.53 ± 0.0 ^a^	24.64 ± 4.7 ^a^	18.37 ± 3.5 ^a^
15	Pelargonidin-3-rutinoside	3.84 ± 0.0 ^a^	n.d.	1.24 ± 0.0 ^b^	n.d.
16	Malvidin-3-galactoside	n.d.	1.71 ± 0.0 ^a^	2.32 ± 0.4 ^a^	2.31 ± 0.4 ^a^
17	Syringic acid	107.15 ± 7.4 ^a^	126.47 ± 0.0 ^a^	122.95 ± 19.9 ^a^	107.15 ± 17.4 ^a^
18	Procyanidin A2	n.d.	n.d.	n.d.	n.d.
19	*p*-Coumaric acid	111.66 ± 2.7 ^a^	157.61 ± 4.1 ^a^	117.98 ± 18.7 ^a^	116.17 ± 21.2 ^a^
20	Ferulic acid	45.29 ± 7.8 ^a^	23.75 ± 3.9 ^b^	20.99 ± 0.0 ^b^	33.69 ± 5.5 ^ab^
21	3,5-Dicaffeoylquinic acid	63.90 ± 11.8 ^a^	31.26 ± 6.5 ^b^	29.73 ± 5.5 ^b^	30.98 ± 5.7 ^b^
22	Rutin	25.69 ± 4.1 ^ac^	38.24 ± 0.2 ^b^	17.17 ± 3.1 ^c^	30.31 ± 1.2 ^ab^
23	Hyperoside	497.40 ± 18.9 ^a^	839.45 ± 94.2 ^ab^	979.33 ± 163.0 ^ab^	1090.40 ± 208.6 ^b^
24	Isoquercitrin	202.77 ± 39.6 ^a^	251.18 ± 26.7 ^a^	223.78 ± 43.1 ^a^	185.26 ± 15.3 ^a^
25	Delphindin-3,5-diglucoside	533.93 ± 99.6 ^a^	603.06 ± 53.7 ^a^	850.65 ± 88.2 ^a^	797.94 ± 153.5 ^a^
26	Phloridzin	12.99 ± 0.7 ^a^	14.44 ± 2.9 ^a^	14.09 ± 2.5 ^a^	13.47 ± 2.5 ^a^
27	Naringin	n.d.	n.d.	n.d.	n.d.
28	Quercitrin	34.49 ± 5.6 ^a^	38.13 ± 7.5 ^a^	34.61 ± 0.8 ^a^	26.98 ± 3.9 ^a^
29	Myricetin	n.d.	n.d.	n.d.	n.d.
30	Kaempferol-3-glucoside	43.50 ± 5.4 ^a^	54.20 ± 1.0 ^a^	57.65 ± 9.2 ^a^	38.75 ± 5.7 ^a^
31	Hesperidin	83.94 ± 6.9 ^a^	94.43 ± 14.8 ^a^	30.86 ^b^	13.58 ^b^
32	Ellagic acid	33,564.02 ± 3008.6 ^ab^	45,469.64 ± 8661.8 ^a^	21,961.44 ± 2646.9 ^b^	18,475.13 ± 1933.3 ^b^
33	Quercetin	14.73025 ± 0.0 ^a^	26.47 ± 4.9 ^b^	12.43 ± 1.9 ^a^	8.75 ± 0.6 ^a^
34	Phloretin	2.54 ± 0.0 ^a^	2.05 ± 0.3 ^a^	n.d.	n.d.
35	Kaempferol	55.19 ± 0.0 ^a^	1597.79 ± 0.0 ^b^	n.d.	n.d.
36	Isorhamnetin	101.06 ± 0.0 ^ab^	126.67 ± 0.8 ^a^	76.40 ± 14.6 ^b^	63.16 ± 12.3 ^b^
TOT		194,060.01 ^a^	262,665.54 ^a^	152,291.70 ^a^	141,080.39 ^a^

* Values are expressed as mean ± standard deviation. ^a–c^ Different letters indicate statistically significative differences by ANOVA test (*p*-value < 0.05). n.d. means not detectable.

**Table 2:** Antioxidant activity of acorn flour extracts: TPC and TFC.

TPC (mg GAE/g)	
20 °C	104.72 * ± 1.26 ^a^
60 °C	103.84 ± 1.94 ^a^
80 °C	105.39 ± 2.74 ^a^
100 °C	103.21 ± 2.24 ^a^
**TFC (mg RE/g)**	
20 °C	4.36 ± 0.01 ^a^
60 °C	6.28 ± 0.09 ^b^
80 °C	5.55 ± 0.02 ^c^
100 °C	3.08 ± 0.12 ^d^

* Values are reported as mean values ± standard deviation. ^a–d^ Different letters indicate statistically significative differences by ANOVA test (*p*-value < 0.05).

**Table 3:** Antioxidant activity of acorn flour extracts: DPPH, ABTS, CUPRAC, FRAP, MCA, and PBD.

DPPH (mg TE/g)	
20 °C	493.17 * ± 1.14 ^a^
60 °C	493.57 ± 1.31 ^a^
80 °C	492.49 ± 1.15 ^a^
100 °C	492.53 ± 0.52 ^a^
**ABTS (mg TE/g)**	
20 °C	911.32 ± 9.63 ^a^
60 °C	964.53 ± 0.67 ^b^
80 °C	937.39 ± 3.45 ^c^
100 °C	796.85 ± 4.33 ^d^
**CUPRAC (mg TE/g)**	
20 °C	781.83 ± 15.88 ^a^
60 °C	951.57 ± 3.28 ^b^
80 °C	861.21 ± 16.36 ^c^
100 °C	700.17 ± 1.46 ^d^
**FRAP (mg TE/g)**	
20 °C	621.97 ± 15.93 ^a^
60 °C	730.48 ± 18.05 ^b^
80 °C	631.97 ± 8.54 ^a^
100 °C	562.16 ± 3.78 ^c^
**Metal chelating (mg EDTAE/g)**	
20 °C	20.64 ± 0.42 ^a^
60 °C	23.49 ± 0.31 ^b^
80 °C	26.97 ± 1.07 ^c^
100 °C	24.70 ± 0.23 ^b^
**Phosphomolybdenum (mmol TE/g)**	
20 °C	4.51 ± 0.14 ^a^
60 °C	5.10 ± 0.03 ^b^
80 °C	4.75 ± 0.11 ^c^
100 °C	4.22 ± 0.04 ^d^

* Values are reported as mean values ± standard deviation. ^a–d^ Different letters indicate statistically significative differences by ANOVA test (*p*-value < 0.05).

**Table 4:** Enzyme inhibitory activities of acorn flour extracts: cholinesterase (AChE), butyrylcholinesterase (BChE), tyrosinase, amylase, and glucosidase inhibition assays.

AChE Inhibition (mg GALAE/g)	
20 °C	1.98 * ± 0.02 ^a^
60 °C	2.09 ± 0.03 ^a^
80 °C	1.72 ± 0.04 ^b^
100 °C	1.62 ± 0.09 ^b^
**BChE inhibition (mg GALAE/g)**	
20 °C	2.57 ± 0.07 ^a^
60 °C	2.41 ± 0.21 ^a^
80 °C	2.21 ± 0.26 ^a^
100 °C	2.39 ± 0.07 ^a^
**Tyrosinase inhibition (mg KAE/g)**	
20 °C	18.01 ± 1.03 ^a^
60 °C	21.64 ± 1.18 ^b^
80 °C	22.66 ± 1.36 ^b^
100 °C	21.83 ± 0.15 ^b^
**Amylase inhibition (mmol ACAE/g)**	
20 °C	0.051 ± 0.01 ^a^
60 °C	0.050 ± 0.00 ^a^
80 °C	0.051 ± 0.01 ^a^
100 °C	0.056 ± 0.01 ^b^
**Glucosidase inhibition (mmol ACAE/g)**	
20 °C	0.962 ± 0.06 ^a^
60 °C	0.986 ± 0.00 ^b^
80 °C	0.977 ± 0.00 ^b^
100 °C	0.930 ± 0.00 ^c^

* Values are reported as mean values ± standard deviation. ^a–c^ Different letters indicate statistically significative differences by ANOVA test (*p*-value < 0.05).

## Data Availability

The data presented in this study are available in the article.

## Supplementary Materials

The following supporting information can be downloaded at: https://www.mdpi.com/article/10.3390/antiox13121526/s1.
