# Supplementary material for: Quantification of Bioactive Compounds by HPLC-ESI-MS/MS and Evaluation of Antioxidant and Enzyme Inhibitory Activities of Acorn Flour Extracts
Source: Antioxidants (Basel). 2024 Dec 13;13(12):1526. doi: 10.3390/antiox13121526 (PMC11727278; doi:10.3390/antiox13121526)
Supplement: Supplementary file 1 [file antioxidants-13-01526-s001.zip › antioxidants-3342000-supplementary.pdf]

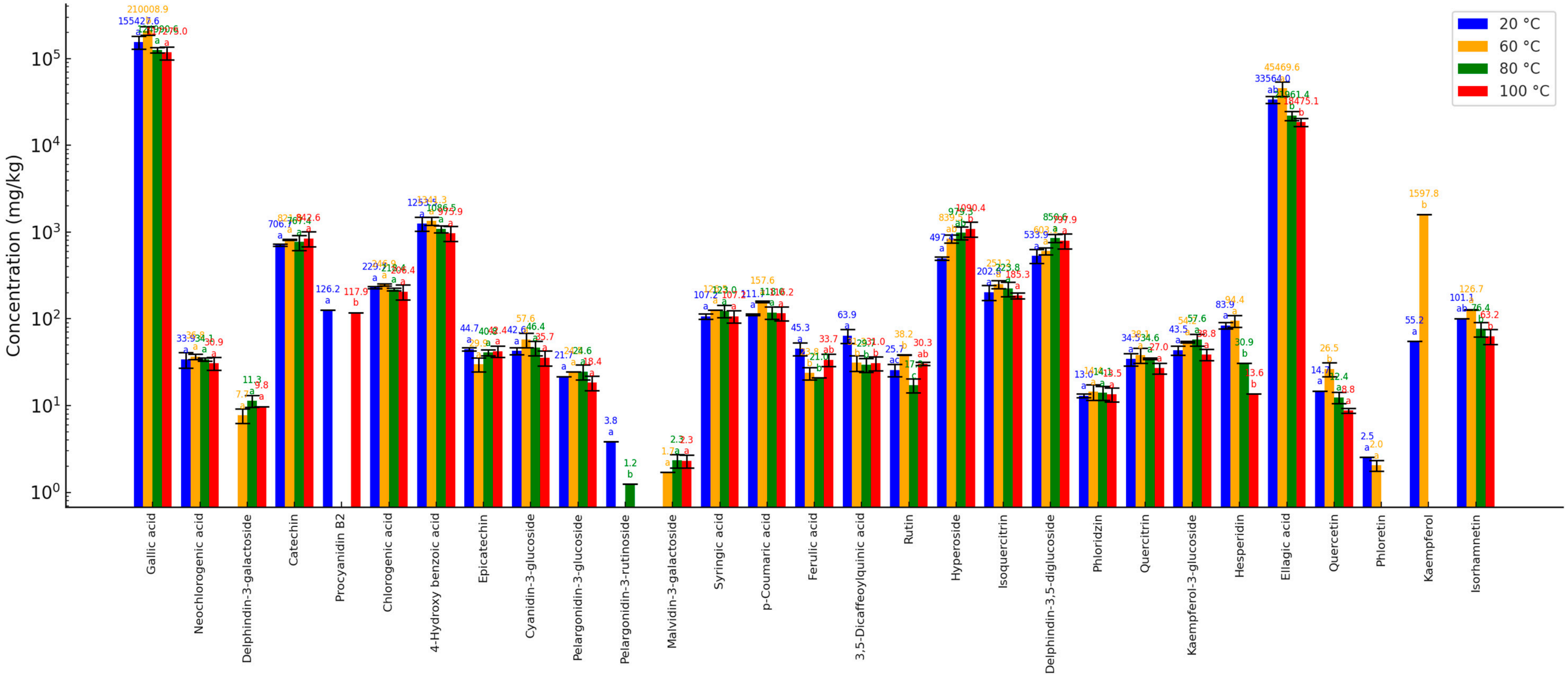


**Figure S1.** HPLC-ESI-MS/MS profiling (mg·kg$^{-1}$) the diversity of bioactive compounds in acorn flour extracts at different temperatures. Color coding corresponds to the extract temperature. Error bars represent standard deviation. Different letters above the bars indicate statistically significant differences between temperatures for each compound by ANOVA test ($p$-value < 0.05).

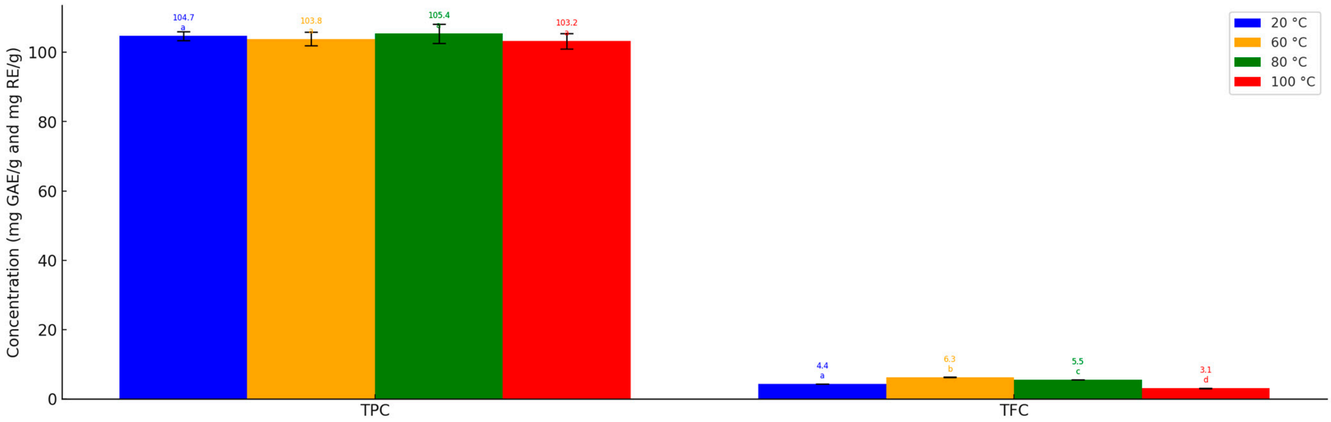


**Figure S2.** Antioxidant assays results displaying antioxidant capacities of acorn flour extracts. The variance in antioxidant activity at each temperature is represented by color-coded bars. **(A)** Total Phenolic Content (TPC) and Total Flavonoid Content (TFC).

Color coding corresponds to the extract temperature. Error bars represent standard deviation. Different letters above the bars indicate statistically significant differences between temperatures for each antioxidant activity by ANOVA test ($p$-value < 0.05). Total Phenolic Content (TPC) is measured in mg GAE/g, Total Flavonoid Content (TFC) is measured in mg RE/g.

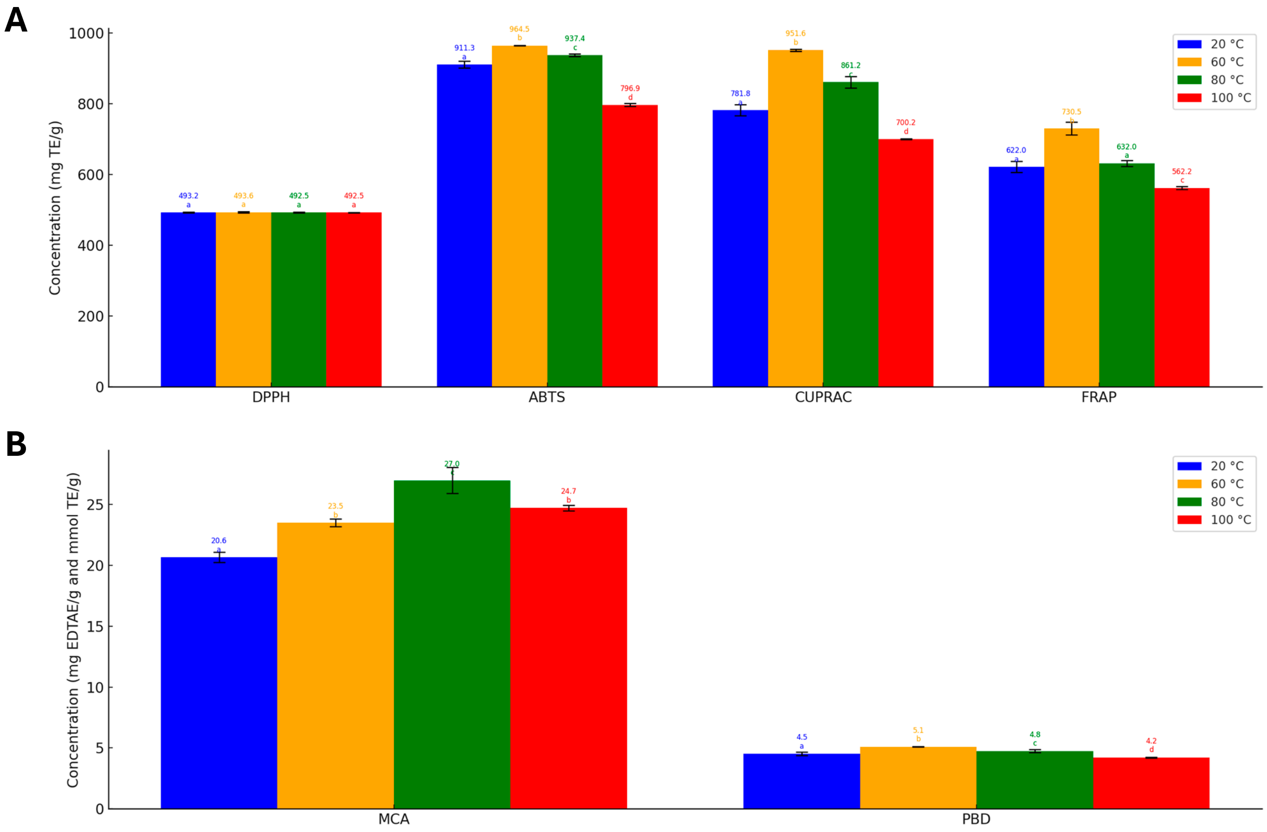


**Figure S3.** Antioxidant assays results displaying antioxidant capacities of acorn flour extracts. The variance in antioxidant activity at each temperature is represented by color-coded bars. **(A)** Antioxidant activities (DPPH, ABTS, CUPRAC, and FRAP). **(B)** Metal chelating and Phosphomolybdenum activities.

Color coding corresponds to the extract temperature. Error bars represent standard deviation. Different letters above the bars indicate statistically significant differences between temperatures for each antioxidant activity by ANOVA test (*p*-value $< 0.05$). Antioxidant activities (DPPH, ABTS, CUPRAC, FRAP) are measured in mg TE/g. Metal chelating activity (MCA) is measured in mg EDTAE/g, and phosphomolybdenum assay (PBD) is measured in mmol TE/g.

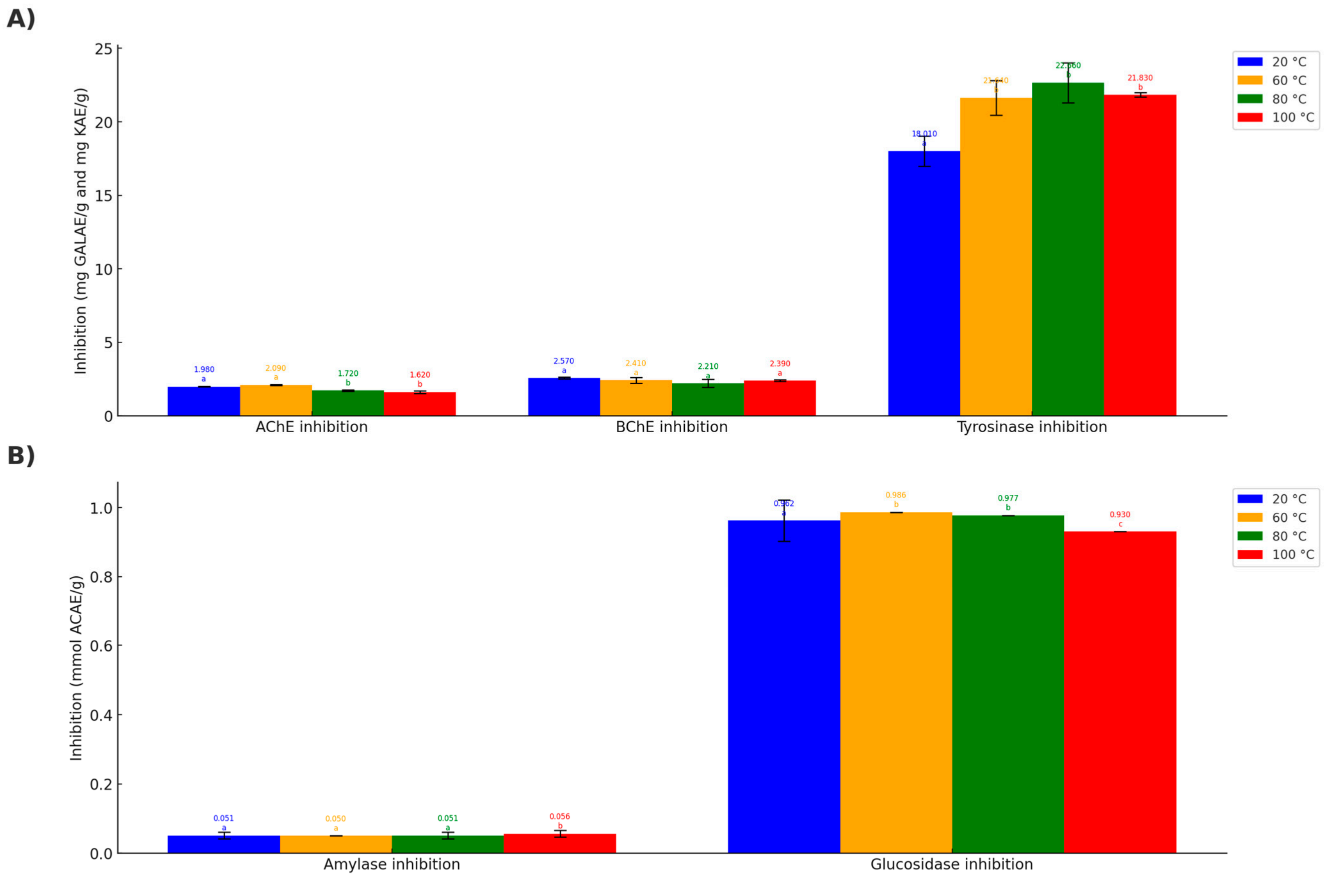


**Figure S4.** Enzyme inhibitory activity profiles of acorn flour extracts across varying temperatures. The efficacy of each extract at different temperatures is color-coded. **(A)** Inhibitory activities of Acetylcholinesterase (AChE), Butyrylcholinesterase (BChE) and Tyrosinase **(B)** Inhibitory activities of Amylase and Glucosidase. * Color bars represent different temperatures. Color coding corresponds to the extract temperature. Error bars represent standard deviation. Different letters above the bars indicate statistically significant differences between temperatures for each enzyme inhibitory activity by ANOVA test (p-value < 0.05). Inhibition is measured in mg GALAE/g for Acetylcholinesterase (AChE) and Butyrylcholinesterase (BChE), mg KAE/g for Tyrosinase, and mmol ACAE/g for Amylase and Glucosidase.

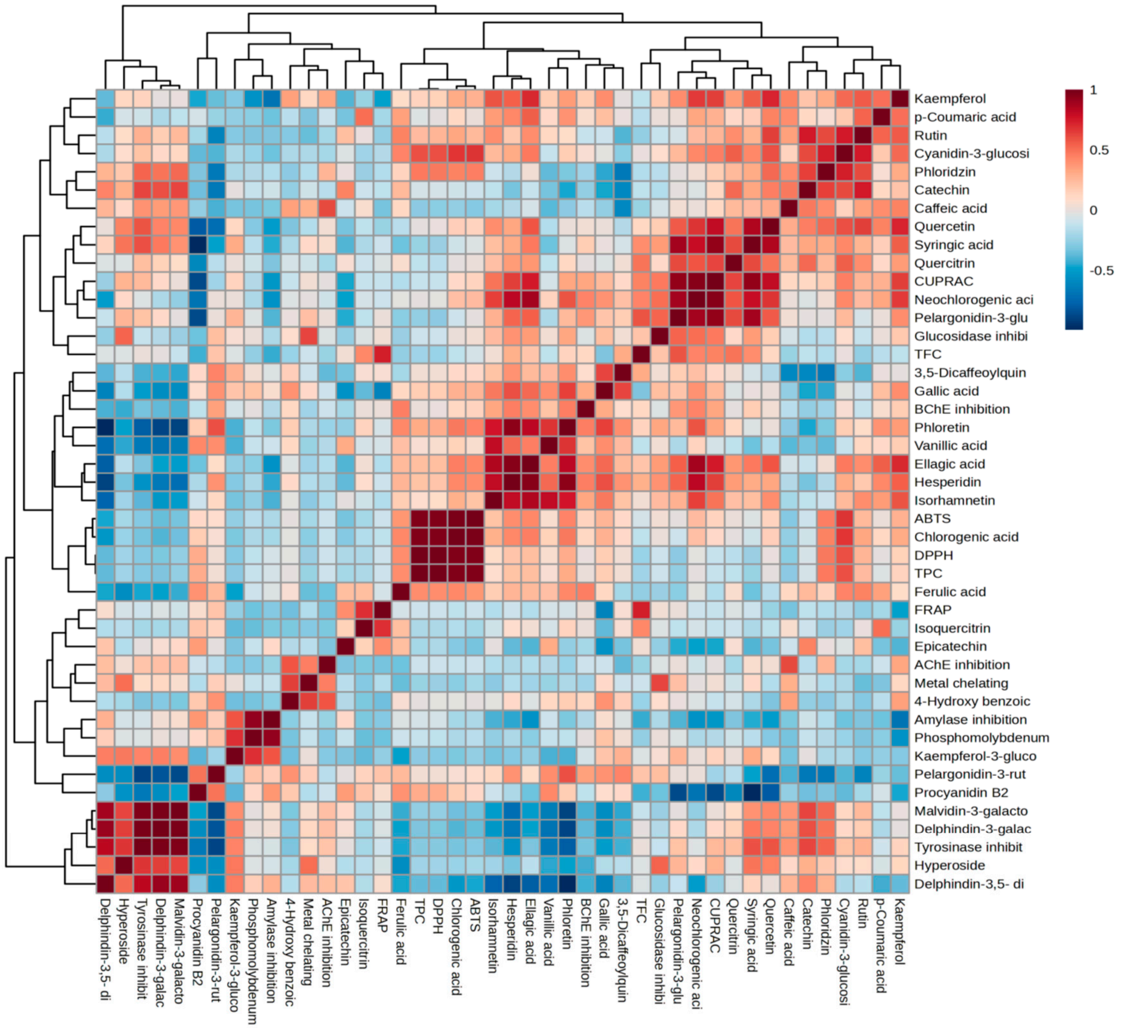


**Figure S5.** Pearson correlation matrix between polyphenol content and antioxidant assays. The heatmap shows the strength of the correlations between individual polyphenols and antioxidant activities assays. The Pearson correlation graphs can be used to identify patterns of positive or negative correlations between different parameters, according to the numbers on the left. The color distribution was fixed (− 1;+1).

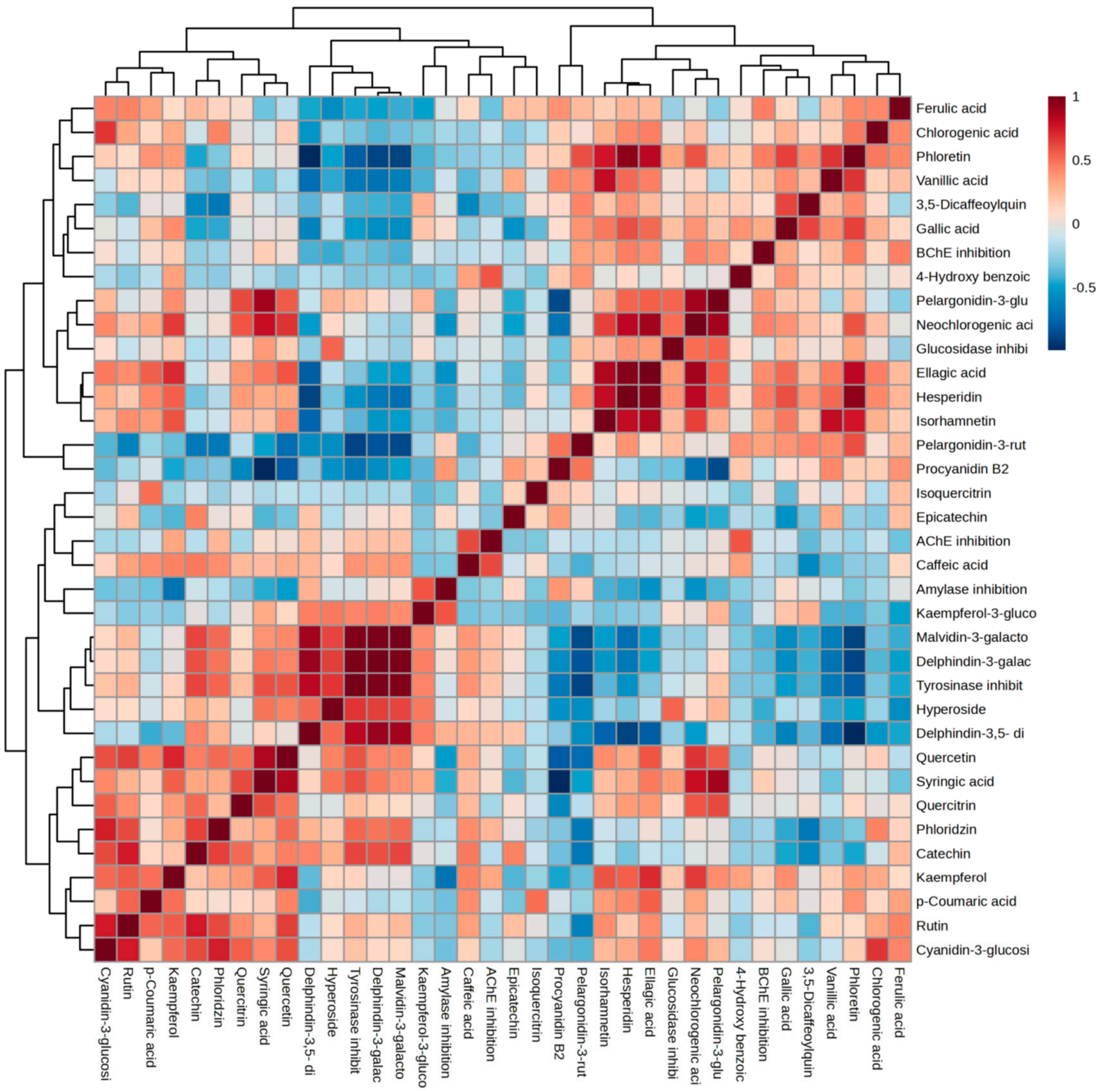


**Figure S6.** Pearson correlation matrix between polyphenol content and enzyme inhibition assays. The heatmap illustrates the relationships between polyphenols and enzyme inhibition efficacy. The Pearson correlation graphs can be used to identify patterns of positive or negative correlations between different parameters, according to the numbers on the left. The color distribution was fixed (− 1;+1).

**Table S1.** HPLC–MS/MS acquisition parameters (dynamic-MRM mode) used for the analysis of the 38 marker compounds.

| No. | Compounds | Precursor ion, *m/z* | Product ion, *m/z* | Fragm-entor, V | Collision energy, V | Polarity | Retention time (Rt, min) | Delta retention time (ΔRt) |
|---|---|---|---|---|---|---|---|---|
| 1 | Gallic acid | 169 | 125.2* | 97 | 12 | Negative | 6.96 | 2 |
| 2 | Neochlorogenic acid | 353 | 191.2*, 179 | 82 | 12, 12 | Negative | 9.52 | 2 |
| 3 | Delphindin-3-galactoside | 465.01 | 303* | 121 | 20 | Positive | 11.36 | 2 |
| 4 | (+)-Catechin | 289 | 245.2*,109.2 | 131 | 8, 20 | Negative | 11.44 | 2 |
| 5 | Procyanidin B2 | 576.99 | 576.99*, 321.2 | 160 | 0, 32 | Negative | 12.41 | 2 |
| 6 | Chlorogenic acid | 353 | 191.2*, 127.5 | 82 | 12, 20 | Negative | 12.42 | 2 |
| 7 | *p*-Hydroxybenzoic acid | 137 | 93.2* | 92 | 16 | Negative | 12.86 | 2 |
| 8 | (-)-Epicatechin | 289 | 245.1*, 109.1 | 126 | 8, 20 | Negative | 13.03 | 2 |
| 9 | Cyanidin-3-glucoside | 449 | 287.3*, 255.6 | 121 | 20, 20 | Positive | 13.14 | 2 |
| 10 | Petunidin-3-glucoside | 479.01 | 317*, 302 | 121 | 20, 44 | Positive | 13.26 | 2 |
| 11 | 3-Hydroxybenzoic acid | 137 | 93.2* | 88 | 8 | Negative | 13.59 | 2 |
| 12 | Caffeic acid | 179 | 135.2*, 134.1 | 92 | 12, 24 | Negative | 13.65 | 2 |
| 13 | Vanillic acid | 167 | 152.4*, 108.1 | 88 | 12, 20 | Negative | 14.32 | 2 |
| 14 | Pelargonidin-3-glucoside | 433.01 | 271*, 121 | 116 | 24, 50 | Positive | 14.52 | 2 |
| 15 | Pelagonidin-3-rutinoside | 579.01 | 271* | 145 | 32 | Positive | 14.56 | 2 |
| 16 | Malvidin-3-galactoside | 493.01 | 331*, 315.1 | 121 | 20, 50 | Positive | 14.64 | 2 |
| 17 | Syringic acid | 196.9 | 182.2*, 121.2 | 93 | 8, 12 | Negative | 15.28 | 2 |
| 18 | Procyanidin A2 | 575 | 575*, 285 | 170 | 0, 20 | Negative | 16.18 | 2 |
| 19 | *p*-Coumaric acid | 163 | 119.2*, 93.2 | 83 | 12, 36 | Negative | 16.70 | 2 |
| 20 | Ferulic acid | 193 | 134.2*, 131.6 | 83 | 12, 8 | Negative | 17.10 | 2 |
| 21 | 3,5-Dicaffeoylquinic acid | 514.9 | 353.1*, 191 | 117 | 8, 28 | Negative | 17.61 | 2 |
| 22 | Rutin | 609 | 300.2*, 271.2 | 170 | 32, 50 | Negative | 17.73 | 2 |
| 23 | Hyperoside | 465.01 | 303*, 61.1 | 97 | 8, 50 | Positive | 18.33 | 2 |
| 24 | Isoquercitrin | 463 | 271.2*, 300.2 | 155 | 44, 24 | Negative | 18.36 | 2 |
| 25 | Delphindin-3,5-diglucoside | 462.9 | 300.1* | 165 | 24 | Negative | 18.38 | 2 |
| 26 | Phloridzin | 435.39 | 273*, 167 | 155 | 8, 28 | Negative | 18.83 | 2 |
| 27 | Quercitrin | 446.99 | 300.2*, 301.2 | 160 | 24, 16 | Negative | 19.61 | 2 |
| 28 | Myricetin | 316.99 | 179.1*, 182 | 150 | 16, 24 | Negative | 19.61 | 2 |
| 29 | Naringin | 578.99 | 271.3*, 151.3 | 170 | 32, 44 | Negative | 19.62 | 2 |
| 30 | Kaempferol-3-glucoside | 447 | 284.2*, 255.2 | 170 | 24, 40 | Negative | 19.77 | 2 |
| 31 | Hesperidin | 611.01 | 303*, 334.8 | 112 | 20, 12 | Positive | 20.19 | 2 |
| 32 | Ellagic acid | 301 | 301*, 229 | 170 | 0, 24 | Negative | 21.41 | 2 |
| 33 | Quercetin | 300.99 | 151.2*, 179.2 | 145 | 16, 12 | Negative | 21.87 | 2 |
| 34 | Phloretin | 272.99 | 167*, 123 | 116 | 8, 20 | Negative | 22.30 | 2 |

| | | | | | | | | |
|---|---|---|---|---|---|---|---|---|
| 35 | Kaempferol | 287.01 | 153*, 69.1 | 60 | 36, 50 | Positive | 23.84 | 2 |
| 36 | Isorhamnetin | 314.99 | 300.2*, 196.1 | 145 | 16, 4 | Negative | 24.57 | 2 |

* These product ions were used for quantification.

**Table S2.** Validation parameters: equation of the calibration curve, linearity, LOQ and LOD.

| **Compounds** | **Equation of the Calibration Curve** | **Linearity ($R^2$)** | **LOQ ($mg \cdot L^{-1}$)** | **LOD ($mg \cdot L^{-1}$)** |
|---|---|---|---|---|
| Gallic acid | $y = 32080x - 168.82$ | $R^2 = 0.9999$ | 0.0051 | 0.0015 |
| Neochlorogenic acid | $y = 12274x - 168.72$ | $R^2 = 0.9992$ | 0.0017 | 0.0005 |
| Delphindin-3-galactoside | $y = 172089x + 4007.1$ | $R^2 = 0.9999$ | 0.0238 | 0.0072 |
| (+)-Catechin | $y = 12748x - 953.78$ | $R^2 = 0.9989$ | 0.0091 | 0.0027 |
| Procyanidin B2 | $y = 14169x - 2853.8$ | $R^2 = 0.9982$ | 0.0800 | 0.0240 |
| Chlorogenic acid | $y = 37476x + 11.196$ | $R^2 = 0.9996$ | 0.0008 | 0.0002 |
| *p*-Hydroxybenzoic acid | $y = 44062x - 4131.3$ | $R^2 = 0.9982$ | 0.0223 | 0.0067 |
| (-)-Epicatechin | $y = 9533.8x + 51.293$ | $R^2 = 0.996$ | 0.0164 | 0.0049 |
| Cyanidin-3-glucoside | $y = 303515x + 9089.9$ | $R^2 = 0.9968$ | 0.0704 | 0.0211 |
| Petunidin-3-glucoside | $y = 393271x + 36058$ | $R^2 = 0.9067$ | 0.0299 | 0.0090 |
| 3-Hydroxybenzoic acid | $y = 41403x - 8683.2$ | $R^2 = 0.9902$ | 0.0189 | 0.0057 |
| Caffeic acid | $y = 55844x - 2788.4$ | $R^2 = 0.9997$ | 0.0305 | 0.0092 |
| Vanillic acid | $y = 2828.2x - 556.13$ | $R^2 = 0.9994$ | 0.0690 | 0.0207 |
| Pelargonidin-3-glucoside | $y = 435793x + 62899$ | $R^2 = 0.8122$ | 0.0217 | 0.0065 |
| Pelagonidin-3-rutinoside | $y = 92730x + 7976.6$ | $R^2 = 0.9233$ | 0.0407 | 0.0122 |
| Malvidin-3-galactoside | $y = 439326x + 35053$ | $R^2 = 0.933$ | 0.0311 | 0.0093 |
| Syringic acid | $y = 3257.8x - 418.25$ | $R^2 = 0.9964$ | 0.0123 | 0.0037 |
| Procyanidin A2 | $y = 30553x - 3175.4$ | $R^2 = 0.9973$ | 0.0495 | 0.0149 |
| *p*-Coumaric acid | $y = 55290x - 511.92$ | $R^2 = 0.9998$ | 0.0060 | 0.0018 |
| Ferulic acid | $y = 15031x - 1052.2$ | $R^2 = 0.9991$ | 0.0147 | 0.0044 |
| 3,5-Dicaffeoylquinic acid | $y = 12819x - 20.082$ | $R^2 = 0.9991$ | 0.0037 | 0.0111 |
| Rutin | $y = 17124x - 80.872$ | $R^2 = 0.9995$ | 0.0016 | 0.0005 |
| Hyperoside | $y = 95098x + 479.28$ | $R^2 = 1$ | 0.0368 | 0.0110 |
| Isoquercitrin | $y = 19532x + 98.459$ | $R^2 = 0.9932$ | 0.0006 | 0.0002 |

| | | | | |
|---|---|---|---|---|
| Delphindin-3,5-diglucoside | y = 23225x - 64.904 | $R^2$ = 0.9999 | 0.0045 | 0.0014 |
| Phloridzin | y = 89837x - 106.72 | $R^2$ = 0.9999 | 0.0004 | 0.0001 |
| Quercitrin | y = 20905x + 400.7 | $R^2$ = 0.9998 | 0.0042 | 0.0013 |
| Myricetin | y = 11087x - 1085.2 | $R^2$ = 0.9991 | 0.0316 | 0.0095 |
| Naringin | y = 6840.9x - 393.15 | $R^2$ = 0.9991 | 0.0027 | 0.0008 |
| Kaempferol-3-glucoside | y = 46030x + 507.81 | $R^2$ = 0.9922 | 0.0019 | 0.0006 |
| Hesperidin | y = 5754.5x + 66.456 | $R^2$ = 0.9952 | 0.1429 | 0.0429 |
| Ellagic acid | y = 69929x - 185.72 | $R^2$ = 0.9998 | 0.0325 | 0.0098 |
| Quercetin | y = 21705x - 4.1377 | $R^2$ = 0.9942 | 0.0054 | 0.0016 |
| Phloretin | y = 125928x + 528.08 | $R^2$ = 0.9996 | 0.0003 | 0.0001 |
| Kaempferol | y = 10782x + 2387 | $R^2$ = 0.7103 | 0.2941 | 0.0883 |
| Isorhamnetin | y = 75989x - 341.83 | $R^2$ = 0.9998 | 0.0006 | 0.0002 |

# Table S3. Pearson correlation coefficients between bioactive compounds and antioxidant assays.

| | Delphindin-3,5- di | Hyperoside | Tyrosinase inhibit | Delphindin-3-galac | Malvidin-3-galacto | Procyanidin B2 | Pelargonidin-3-rut | Kaempferol-3-gluco | Phosphomolybdenum | Amylase inhibition | 4-Hydroxybenzoic | Metal chelating | AChE inhibition | Epicatechin | Isoquercitrin |
|---|---|---|---|---|---|---|---|---|---|---|---|---|---|---|---|
| Delphindin-3,5- di | 1 | 0.51409 | 0.81456 | 0.90396 | 0.88908 | -0.27345 | -0.55562 | 0.46238 | 0.1546 | 0.28089 | -0.1509 | 0.23447 | 0.22837 | 0.19566 | -0.16546 |
| Hyperoside | 0.51409 | 1 | 0.66931 | 0.64657 | 0.62569 | -0.53498 | -0.5567 | 0.47225 | -0.02796 | -0.05759 | -0.19436 | 0.50784 | 0.03256 | -0.15259 | -0.14581 |
| Tyrosinase inhibit | 0.81456 | 0.66931 | 1 | 0.98419 | 0.97302 | -0.66747 | -0.87963 | 0.43597 | -0.04697 | -0.05708 | -0.3081 | 0.08649 | 0.20602 | -0.01593 | -0.2034 |
| Delphindin-3-galac | 0.90396 | 0.64657 | 0.98419 | 1 | 0.98966 | -0.56497 | -0.82336 | 0.45543 | 0.0091 | 0.04502 | -0.27581 | 0.12792 | 0.22188 | 0.05284 | -0.19761 |
| Malvidin-3-galacto | 0.88908 | 0.62569 | 0.97302 | 0.98966 | 1 | -0.47913 | -0.87145 | 0.40733 | -0.01414 | 0.06173 | -0.29935 | 0.06941 | 0.21601 | 0.09375 | -0.1848 |
| Procyanidin B2 | -0.27345 | -0.53498 | -0.66747 | -0.56497 | -0.47913 | 1 | 0.49202 | -0.37897 | 0.09253 | 0.37925 | 0.18326 | -0.15555 | -0.10126 | 0.35669 | 0.19428 |
| Pelargonidin-3-rut | -0.55562 | -0.5567 | -0.87963 | -0.82336 | -0.87145 | 0.49202 | 1 | -0.21024 | 0.19988 | 0.15886 | 0.38724 | 0.15773 | -0.14861 | -0.00302 | 0.13288 |
| Kaempferol-3-gluco | 0.46238 | 0.47225 | 0.43597 | 0.45543 | 0.40733 | -0.37897 | -0.21024 | 1 | 0.69109 | 0.57668 | -0.34327 | 0.01977 | -0.27905 | -0.30919 | -0.35473 |
| Phosphomolybdenum | 0.1546 | -0.02796 | -0.04697 | 0.0091 | -0.01414 | 0.09253 | 0.19988 | 0.69109 | 1 | 0.92127 | -0.32193 | -0.11671 | -0.28038 | -0.10882 | -0.34874 |
| Amylase inhibition | 0.28089 | -0.05759 | -0.05708 | 0.04502 | 0.06173 | 0.37925 | 0.15886 | 0.57668 | 0.92127 | 1 | -0.26693 | -0.11311 | -0.23347 | 0.09619 | -0.29684 |
| 4-Hydroxybenzoic | -0.1509 | -0.19436 | -0.3081 | -0.27581 | -0.29935 | 0.18326 | 0.38724 | -0.34327 | -0.32193 | -0.26693 | 1 | 0.64269 | 0.57497 | -0.16637 | -0.29164 |
| Metal chelating | 0.23447 | 0.50784 | 0.08649 | 0.12792 | 0.06941 | -0.15555 | 0.15773 | 0.01977 | -0.11671 | -0.11311 | 0.64269 | 1 | 0.45943 | -0.1375 | -0.27848 |
| AChE inhibition | 0.22837 | 0.03256 | 0.20602 | 0.22188 | 0.21601 | -0.10126 | -0.14861 | -0.27905 | -0.28038 | -0.23347 | 0.57497 | 0.45943 | 1 | -0.30911 | -0.29584 |
| Epicatechin | 0.19566 | -0.15259 | -0.01593 | 0.05284 | 0.09375 | 0.35669 | -0.00302 | -0.30919 | -0.10882 | 0.09619 | -0.16637 | -0.1375 | -0.30911 | 1 | 0.14569 |
| Isoquercitrin | -0.16546 | -0.14581 | -0.2034 | -0.19761 | -0.1848 | 0.19428 | 0.13288 | -0.35473 | -0.34874 | -0.29684 | -0.29164 | -0.27848 | -0.29584 | 0.14569 | 1 |
| FRAP | 0.06779 | -0.04976 | -0.08644 | -0.04537 | -0.07357 | 0.07049 | 0.20344 | -0.24971 | -0.33288 | -0.28147 | -0.34237 | -0.16022 | -0.31635 | 0.40773 | 0.69228 |
| Ferulic acid | -0.46801 | -0.57344 | -0.45568 | -0.47273 | -0.4246 | 0.39807 | 0.2383 | -0.47727 | -0.08388 | -0.03749 | 0.05872 | -0.34754 | -0.33498 | 0.22835 | 0.22424 |
| TPC | -0.35559 | -0.21671 | -0.28087 | -0.30893 | -0.2525 | 0.2934 | 0.04825 | -0.27186 | -0.11862 | -0.06058 | -0.01446 | -0.22844 | -0.08726 | -0.19429 | -0.18463 |
| DPPH | -0.35639 | -0.21547 | -0.27526 | -0.30461 | -0.24455 | 0.30002 | 0.03099 | -279 | -0.12628 | -0.06297 | -0.02226 | -0.24072 | -0.08743 | -0.18692 | -0.17916 |
| Chlorogenic acid | -0.51553 | -0.22047 | -0.31839 | -0.38666 | -0.34019 | 0.16334 | 0.05342 | -0.30329 | -177 | -0.19639 | -0.02579 | -0.26005 | -0.11625 | -0.28264 | -0.16327 |
| ABTS | -0.45953 | -0.18967 | -0.28605 | -0.34759 | -0.31695 | 0.10476 | 0.0796 | -0.24777 | -0.14415 | -0.18301 | -0.00841 | -0.2011 | -0.10237 | -0.31419 | -0.2021 |
| Isorhamnetin | -0.76843 | -0.2185 | -0.38187 | -0.51461 | -0.49677 | -0.09459 | 0.10848 | -0.32258 | -0.24823 | -0.40727 | -0.02291 | -0.26769 | -0.15811 | -0.01137 | -0.07392 |
| Hesperidin | -0.89218 | -316 | -0.54594 | -0.67861 | -0.71034 | -0.17905 | 0.38828 | -0.28519 | -0.17964 | -0.44862 | 0.09103 | -0.16547 | -0.18704 | -0.36604 | 0.07827 |
| Ellagic acid | -0.7949 | -0.13992 | -0.31192 | -0.47445 | -0.48782 | -0.33239 | 0.08635 | -0.25486 | -0.26688 | -0.53405 | -0.03239 | -0.21904 | -0.15695 | -0.38671 | 0.04626 |
| Vanillic acid | -0.71638 | -0.45125 | -0.65967 | -0.70014 | -0.65494 | 0.4305 | 0.40983 | -0.41366 | -0.10628 | -0.08843 | 0.11041 | -0.23402 | -0.16924 | 0.31406 | -0.05125 |
| Phloretin | -0.98986 | -0.48326 | -0.79471 | -0.88863 | -0.89393 | 0.17378 | 0.58957 | -0.40859 | -0.13235 | -0.30312 | 0.16786 | -0.17978 | -0.22533 | -0.24422 | 0.14384 |
| BChE inhibition | -0.40989 | -0.42985 | -0.31915 | -366 | -0.41308 | -0.12396 | 0.35326 | -0.06654 | -0.00379 | -171 | 0.11978 | -0.21003 | -0.08983 | -0.20139 | 0.01725 |
| Gallic acid | -0.62031 | -0.15869 | -0.49769 | -0.55858 | -0.5679 | 0.07833 | 0.39473 | 0.19537 | 0.21448 | 73 | 0.40082 | 0.09619 | -0.08687 | -0.54388 | -0.36436 |
| 3,5-Dicaffeoylquin | -0.37717 | -0.1436 | -0.40923 | -0.42126 | -0.45337 | 0.09239 | 0.43921 | 0.28397 | 0.03807 | -0.04148 | 0.15808 | -0.08471 | -0.35896 | -0.32214 | 0.05538 |
| TFC | -0.04305 | -0.0264 | 0.01211 | -0.0143 | -0.10719 | -0.41976 | 0.20525 | -0.04053 | -0.24904 | -0.4332 | -0.22729 | -0.08805 | -0.23509 | 0.0429 | 0.43164 |
| Glucosidase inhibi | -0.24688 | 0.53506 | -0.11849 | -0.17128 | -0.2368 | -0.33306 | 0.22962 | 0.05559 | 0.00726 | -0.18197 | 0.08211 | 0.63046 | -0.03871 | -0.24517 | -0.03085 |
| Pelargonidin-3-glu | -0.1116 | 0.26121 | 0.2081 | 0.10501 | -0.01389 | -0.85818 | 0.01074 | 0.25985 | -0.03056 | -0.39657 | 0.00262 | 0.21898 | 0.00798 | -0.43819 | -0.13256 |
| Neochlorogenic aci | -0.49722 | 0.09066 | -0.02627 | -0.18013 | -0.25332 | -0.69651 | 0.0163 | 0.01055 | -0.17737 | -0.53648 | -0.03231 | -0.00706 | -0.0803 | -0.46988 | -0.05515 |
| CUPRAC | -0.24238 | 0.27281 | 0.25008 | 0.10061 | 0.02138 | -0.86692 | -0.20608 | 0.14329 | -0.17244 | -0.52743 | -0.10563 | 0.03676 | -0.01632 | -0.46133 | -0.11033 |
| Quercitrin | -0.04654 | -0.03237 | 0.22894 | 0.14733 | 0.08422 | -0.59668 | -0.14425 | 0.00746 | -0.07475 | -0.31228 | -0.0626 | -0.07784 | -0.19482 | 0.05625 | -0.09204 |
| Syringic acid | 0.13588 | 0.48598 | 0.58909 | 0.46596 | 0.38682 | -0.98834 | -0.47065 | 0.31184 | -0.13619 | -0.44214 | -0.18668 | 0.1003 | 0.07107 | -0.39133 | -0.17526 |
| Quercetin | 0.0111 | 0.44061 | 0.58803 | 0.43552 | 0.4284 | -0.80305 | -0.71794 | 0.1218 | -0.2887 | -0.49938 | -0.30975 | -0.15529 | 0.04033 | -0.31825 | -0.1222 |
| Caffeic acid | 0.26545 | 0.09154 | 0.38858 | 0.36953 | 0.37765 | -0.24905 | -0.4073 | -0.32721 | -0.31691 | -0.30062 | 0.34284 | 0.29245 | 0.6168 | -0.09346 | 0.06129 |
| Catechin | 0.43487 | 0.28667 | 0.62645 | 0.59758 | 0.62532 | -0.34272 | -0.67759 | -0.01351 | -0.1129 | -0.08005 | -0.25687 | -0.0768 | -0.14678 | 0.44182 | -0.10036 |
| Phloridzin | 0.27069 | 0.17702 | 0.53308 | 0.48092 | 0.52177 | -0.30965 | -0.6687 | -0.18589 | -0.17303 | -0.16503 | -0.26796 | -0.15755 | 0.25844 | -0.00102 | -0.24134 |
| Cyanidin-3-glucosi | -0.17437 | 0.07967 | 0.20872 | 0.10233 | 0.11925 | -0.36163 | -0.38443 | -0.18438 | -0.2244 | -0.33649 | -0.18327 | -0.18819 | -0.2041 | -0.0354 | -0.2143 |
| Rutin | -0.14009 | 0.08352 | 0.27987 | 0.17189 | 0.2454 | -0.19654 | -0.61236 | -0.28377 | -0.30743 | -0.31168 | -0.28278 | -0.35239 | -0.20241 | 0.22485 | -0.00205 |
| p-Coumaric acid | -0.42965 | -0.05517 | -0.07851 | -0.18268 | -0.13253 | -0.05855 | -0.23205 | -0.24935 | -0.26276 | -0.33074 | -0.14157 | -0.31323 | -0.09042 | -0.3161 | 0.50633 |
| Kaempferol | -0.35763 | 0.1262 | 0.13405 | -0.00976 | -0.00169 | -0.45801 | -0.34546 | -0.28796 | -0.54472 | -0.68899 | 0.34871 | 0.12362 | 0.32666 | -0.38964 | -0.23309 |

| | FRAP | Ferulic acid | TPC | DPPH | Chlorogenic acid | ABTS | Isorhamnetin | Hesperidin | Ellagic acid | Vanillic acid | Phloretin | BChE inhibition | Gallic acid | 3,5-Dicaffeoylquin | TFC |
|---|---|---|---|---|---|---|---|---|---|---|---|---|---|---|---|
| Delphindin-3,5- di | 0.06779 | -0.46801 | -0.35559 | -0.35639 | -0.51553 | -0.45953 | -0.76843 | -0.89218 | -0.7949 | -0.71638 | -0.98986 | -0.40989 | -0.62031 | -0.37717 | -0.04305 |
| Hyperoside | -0.04976 | -0.57344 | -0.21671 | -0.21547 | -0.22047 | -0.18967 | -0.2185 | -316 | -0.13992 | -0.45125 | -0.48326 | -0.42985 | -0.15869 | -0.1436 | -0.0264 |
| Tyrosinase inhibit | -0.08644 | -0.45568 | -0.28087 | -0.27526 | -0.31839 | -0.28605 | -0.38187 | -0.54594 | -0.31192 | -0.65967 | -0.79471 | -0.31915 | -0.49769 | -0.40923 | 0.01211 |
| Delphindin-3-galac | -0.04537 | -0.47273 | -0.30893 | -0.30461 | -0.38666 | -0.34759 | -0.51461 | -0.67861 | -0.47445 | -0.70014 | -0.88863 | -366 | -0.55858 | -0.42126 | -0.0143 |
| Malvidin-3-galacto | -0.07357 | -0.4246 | -0.2525 | -0.24455 | -0.34019 | -0.31695 | -0.49677 | -0.71034 | -0.48782 | -0.65494 | -0.89393 | -0.41308 | -0.5679 | -0.45337 | -0.10719 |
| Procyanidin B2 | 0.07049 | 0.39807 | 0.2934 | 0.30002 | 0.16334 | 0.10476 | -0.09459 | -0.17905 | -0.33239 | 0.4305 | 0.17378 | -0.12396 | 0.07833 | 0.09239 | -0.41976 |
| Pelargonidin-3-rut | 0.20344 | 0.2383 | 0.04825 | 0.03099 | 0.05342 | 0.0796 | 0.10848 | 0.38828 | 0.08635 | 0.40983 | 0.58957 | 0.35326 | 0.39473 | 0.43921 | 0.20525 |
| Kaempferol-3-gluco | -0.24971 | -0.47727 | -0.27186 | -279 | -0.30329 | -0.24777 | -0.32258 | -0.28519 | -0.25486 | -0.41366 | -0.40859 | -0.06654 | 0.19537 | 0.28397 | -0.04053 |
| Phosphomolybdenum | -0.33288 | -0.08388 | -0.11862 | -0.12628 | -177 | -0.14415 | -0.24823 | -0.17964 | -0.26688 | -0.10628 | -0.13235 | -0.00379 | 0.21448 | 0.03807 | -0.24904 |
| Amylase inhibition | -0.28147 | -0.03749 | -0.06058 | -0.06297 | -0.19639 | -0.18301 | -0.40727 | -0.44862 | -0.53405 | -0.08843 | -0.30312 | -171 | 73 | -0.04148 | -0.4332 |
| 4-Hydroxybenzoic | -0.34237 | 0.05872 | -0.01446 | -0.02226 | -0.02579 | -0.00841 | -0.02291 | 0.09103 | -0.03239 | 0.11041 | 0.16786 | 0.11978 | 0.40082 | 0.15808 | -0.22729 |
| Metal chelating | -0.16022 | -0.34754 | -0.22844 | -0.24072 | -0.26005 | -0.2011 | -0.26769 | -0.16547 | -0.21904 | -0.23402 | -0.17978 | -0.21003 | 0.09619 | -0.08471 | -0.08805 |
| AChE inhibition | -0.31635 | -0.33498 | -0.08726 | -0.08743 | -0.11625 | -0.10237 | -0.15811 | -0.18704 | -0.15695 | -0.16924 | -0.22533 | -0.08983 | -0.08687 | -0.35896 | -0.23509 |
| Epicatechin | 0.40773 | 0.22835 | -0.19429 | -0.18692 | -0.28264 | -0.31419 | -0.01137 | -0.36604 | -0.38671 | 0.31406 | -0.24422 | -0.20139 | -0.54388 | -0.32214 | 0.0429 |
| Isoquercitrin | 0.69228 | 0.22424 | -0.18463 | -0.17916 | -0.16327 | -0.2021 | -0.07392 | 0.07827 | 0.04626 | -0.05125 | 0.14384 | 0.01725 | -0.36436 | 0.05538 | 0.43164 |
| FRAP | 1 | -0.05022 | -0.08685 | -0.09357 | -0.12164 | -0.09503 | -0.15794 | -0.08362 | -0.1632 | -0.05398 | -0.05229 | 0.02208 | -0.61156 | 0.05849 | 0.74008 |
| Ferulic acid | -0.05022 | 1 | 0.40512 | 0.40986 | 0.42271 | 0.37847 | 0.16443 | 0.2654 | 0.24407 | 0.21875 | 419 | 0.45407 | 0.10633 | -0.20454 | -0.25631 |
| TPC | -0.08685 | 0.40512 | 1 | 0.99956 | 0.96816 | 0.96661 | 0.08157 | 0.20876 | 0.22602 | 0.05551 | 0.30204 | 0.01272 | 0.1691 | 0.04126 | -0.20925 |
| DPPH | -0.09357 | 0.40986 | 0.99956 | 1 | 0.96813 | 0.96291 | 0.09016 | 0.20486 | 0.22829 | 0.06404 | 0.2995 | 0.00389 | 0.16592 | 0.03324 | -0.22413 |
| Chlorogenic acid | -0.12164 | 0.42271 | 0.96816 | 0.96813 | 1 | 0.99057 | 0.28561 | 0.42924 | 0.46179 | 0.15093 | 0.47593 | 0.11519 | 0.2825 | 0.09548 | -0.13313 |
| ABTS | -0.09503 | 0.37847 | 0.96661 | 0.96291 | 0.99057 | 1 | 0.21426 | 0.40527 | 0.42317 | 0.06898 | 0.43474 | 0.1307 | 0.26265 | 0.1048 | -0.07137 |
| Isorhamnetin | -0.15794 | 0.16443 | 0.08157 | 0.09016 | 0.28561 | 0.21426 | 1 | 0.80732 | 0.84809 | 0.81285 | 765 | 0.31769 | 0.47663 | 0.20855 | 0.05131 |
| Hesperidin | -0.08362 | 0.2654 | 0.20876 | 0.20486 | 0.42924 | 0.40527 | 0.80732 | 1 | 0.94754 | 0.52551 | 0.93382 | 0.45748 | 0.60232 | 0.39443 | 0.29188 |
| Ellagic acid | -0.1632 | 0.24407 | 0.22602 | 0.22829 | 0.46179 | 0.42317 | 0.84809 | 0.94754 | 1 | 0.45063 | 0.82408 | 0.40864 | 0.52505 | 0.24351 | 0.1986 |
| Vanillic acid | -0.05398 | 0.21875 | 0.05551 | 0.06404 | 0.15093 | 0.06898 | 0.81285 | 0.52551 | 0.45063 | 1 | 0.67448 | 0.20904 | 0.4102 | 0.23652 | -0.12305 |
| Phloretin | -0.05229 | 419 | 0.30204 | 0.2995 | 0.47593 | 0.43474 | 765 | 0.93382 | 0.82408 | 0.67448 | 1 | 0.45869 | 0.63853 | 0.40809 | 0.13039 |
| BChE inhibition | 0.02208 | 0.45407 | 0.01272 | 0.00389 | 0.11519 | 0.1307 | 0.31769 | 0.45748 | 0.40864 | 0.20904 | 0.45869 | 1 | 0.31473 | 0.07524 | 0.10736 |
| Gallic acid | -0.61156 | 0.10633 | 0.1691 | 0.16592 | 0.2825 | 0.26265 | 0.47663 | 0.60232 | 0.52505 | 0.4102 | 0.63853 | 0.31473 | 1 | 0.6223 | -0.30859 |
| 3,5-Dicaffeoylquin | 0.05849 | -0.20454 | 0.04126 | 0.03324 | 0.09548 | 0.1048 | 0.20855 | 0.39443 | 0.24351 | 0.23652 | 0.40809 | 0.07524 | 0.6223 | 1 | 0.30107 |
| TFC | 0.74008 | -0.25631 | -0.20925 | -0.22413 | -0.13313 | -0.07137 | 0.05131 | 0.29188 | 0.1986 | -0.12305 | 0.13039 | 0.10736 | -0.30859 | 0.30107 | 1 |
| Glucosidase inhibi | 0.03153 | -0.2374 | -0.09076 | -0.10191 | 0.01554 | 0.05139 | 0.23459 | 0.39083 | 0.36946 | 0.0441 | 0.3182 | -0.04036 | 0.22922 | 0.02931 | 0.20265 |
| Pelargonidin-3-glu | 0.00693 | -0.26612 | -0.25642 | -0.27223 | -0.08127 | -0.01036 | 0.27814 | 0.52914 | 0.54224 | -0.18145 | 0.24012 | 0.37572 | 0.19858 | 0.16564 | 0.58317 |
| Neochlorogenic aci | -0.0985 | -0.02062 | -0.01768 | -0.02467 | 0.2166 | 0.23363 | 0.6402 | 0.82799 | 0.87744 | 0.14004 | 0.5855 | 0.43348 | 0.39914 | 0.21667 | 0.43554 |
| CUPRAC | -0.10544 | -0.15949 | -0.11256 | -0.1189 | 0.10142 | 0.13178 | 0.49279 | 0.64309 | 0.74655 | -0.06327 | 0.33796 | 0.33828 | 0.24285 | 0.10323 | 0.44698 |
| Quercitrin | -0.04225 | 0.07422 | -0.13379 | -0.13982 | -0.01373 | 0.01915 | 0.21678 | 0.36525 | 0.38448 | -0.12824 | 0.11703 | -0.01694 | -0.03646 | 0.0286 | 0.4885 |
| Syringic acid | -0.09945 | -0.33172 | -0.23218 | -0.23726 | -0.07365 | -0.02745 | 0.22574 | 0.30976 | 0.46949 | -0.33216 | -0.04092 | 0.16992 | 0.0034 | -0.05866 | 0.41003 |
| Quercetin | -0.23033 | -0.15015 | -0.01023 | -0.00097 | 0.15289 | 0.13692 | 0.40831 | 0.32038 | 0.57957 | -0.15449 | 0.01734 | 43 | 0.01145 | -0.16599 | 0.12147 |
| Caffeic acid | -0.3271 | 0.10692 | -0.27368 | -0.2653 | -0.25884 | -0.2769 | -0.19671 | -0.17264 | -0.05413 | -0.37139 | -0.27183 | -0.14194 | -0.25395 | -0.58989 | -0.2335 |
| Catechin | -0.12223 | 0.25338 | -0.06378 | -0.05314 | -0.07891 | -0.08982 | -0.13076 | -0.31568 | -0.11698 | -0.32081 | -0.45399 | -0.25372 | -0.46703 | -0.58813 | -0.12668 |
| Phloridzin | -0.16331 | 0.13569 | 0.4673 | 0.47483 | 0.44775 | 0.45186 | -0.09681 | -0.17044 | 0.04128 | -0.36206 | -0.30236 | -0.21519 | -0.44686 | -0.67881 | -0.16175 |
| Cyanidin-3-glucosi | -0.17316 | 0.43952 | 0.59524 | 0.59774 | 0.67235 | 0.67593 | 0.25308 | 0.31461 | 0.47135 | -0.11784 | 0.17154 | 0.05189 | -0.01905 | -0.26578 | -0.00043 |
| Rutin | -0.25465 | 0.4465 | 0.23616 | 0.25604 | 0.32381 | 0.24888 | 0.40659 | 0.18108 | 0.41432 | 0.12207 | 0.08309 | -0.10899 | -0.09817 | -0.3941 | -0.24731 |
| p-Coumaric acid | -0.19499 | 0.34491 | -0.02971 | -0.01214 | 0.11053 | 0.01961 | 0.36689 | 0.42939 | 0.55554 | 0.09067 | 0.39002 | 0.06539 | 0.22506 | -0.00151 | -0.14355 |
| Kaempferol | -0.47843 | 0.08949 | 0.14222 | 0.15071 | 0.31572 | 0.27775 | 0.58249 | 0.54434 | 0.70651 | 0.16088 | 0.36709 | 161 | 0.40161 | -0.00542 | -0.12964 |

| | Glucosidase inhibi | Pelargonidin-3-glu | Neochlorogenic aci | CUPRAC | Quercitrin | Syringic acid | Quercetin | Caffeic acid | Catechin | Phloridzin | Cyanidin-3-glucosi | Rutin | p-Coumaric acid | Kaempferol |
|---|---|---|---|---|---|---|---|---|---|---|---|---|---|---|
| Delphindin-3,5- di | -0.24688 | -0.1116 | -0.49722 | -0.24238 | -0.04654 | 0.13588 | 0.0111 | 0.26545 | 0.43487 | 0.27069 | -0.17437 | -0.14009 | -0.42965 | -0.35763 |
| Hyperoside | 0.53506 | 0.26121 | 0.09066 | 0.27281 | -0.03237 | 0.48598 | 0.44061 | 0.09154 | 0.28667 | 0.17702 | 0.07967 | 0.08352 | -0.05517 | 0.1262 |
| Tyrosinase inhibit | -0.11849 | 0.2081 | -0.02627 | 0.25008 | 0.22894 | 0.58909 | 0.58803 | 0.38858 | 0.62645 | 0.53308 | 0.20872 | 0.27987 | -0.07851 | 0.13405 |
| Delphindin-3-galac | -0.17128 | 0.10501 | -0.18013 | 0.10061 | 0.14733 | 0.46596 | 0.43552 | 0.36953 | 0.59758 | 0.48092 | 0.10233 | 0.17189 | -0.18268 | -0.00976 |
| Malvidin-3-galacto | -0.2368 | -0.01389 | -0.25332 | 0.02138 | 0.08422 | 0.38682 | 0.4284 | 0.37765 | 0.62532 | 0.52177 | 0.11925 | 0.2454 | -0.13253 | -0.00169 |
| Procyanidin B2 | -0.33306 | -0.85818 | -0.69651 | -0.86692 | -0.59668 | -0.98834 | -0.80305 | -0.24905 | -0.34272 | -0.30965 | -0.36163 | -0.19654 | -0.05855 | -0.45801 |
| Pelargonidin-3-rut | 0.22962 | 0.01074 | 0.0163 | -0.20608 | -0.14425 | -0.47065 | -0.71794 | -0.4073 | -0.67759 | -0.6687 | -0.38443 | -0.61236 | -0.23205 | -0.34546 |
| Kaempferol-3-gluco | 0.05559 | 0.25985 | 0.01055 | 0.14329 | 0.00746 | 0.31184 | 0.1218 | -0.32721 | -0.01351 | -0.18589 | -0.18438 | -0.28377 | -0.24935 | -0.28796 |
| Phosphomolybdenum | 0.00726 | -0.03056 | -0.17737 | -0.17244 | -0.07475 | -0.13619 | -0.2887 | -0.31691 | -0.1129 | -0.17303 | -0.2244 | -0.30743 | -0.26276 | -0.54472 |
| Amylase inhibition | -0.18197 | -0.39657 | -0.53648 | -0.52743 | -0.31228 | -0.44214 | -0.49938 | -0.30062 | -0.08005 | -0.16503 | -0.33649 | -0.31168 | -0.33074 | -0.68899 |
| 4-Hydroxybenzoic | 0.08211 | 0.00262 | -0.03231 | -0.10563 | -0.0626 | -0.18668 | -0.30975 | 0.34284 | -0.25687 | -0.26796 | -0.18327 | -0.28278 | -0.14157 | 0.34871 |
| Metal chelating | 0.63046 | 0.21898 | -0.00706 | 0.03676 | -0.07784 | 0.1003 | -0.15529 | 0.29245 | -0.0768 | -0.15755 | -0.18819 | -0.35239 | -0.31323 | 0.12362 |
| AChE inhibition | -0.03871 | 0.00798 | -0.0803 | -0.01632 | -0.19482 | 0.07107 | 0.04033 | 0.6168 | -0.14678 | 0.25844 | -0.2041 | -0.20241 | -0.09042 | 0.32666 |
| Epicatechin | -0.24517 | -0.43819 | -0.46988 | -0.46133 | 0.05625 | -0.39133 | -0.31825 | -0.09346 | 0.44182 | -0.00102 | -0.0354 | 0.22485 | -0.3161 | -0.38964 |
| Isoquercitrin | -0.03085 | -0.13256 | -0.05515 | -0.11033 | -0.09204 | -0.17526 | -0.1222 | 0.06129 | -0.10036 | -0.24134 | -0.2143 | -0.00205 | 0.50633 | -0.23309 |
| FRAP | 0.03153 | 0.00693 | -0.0985 | -0.10544 | -0.04225 | -0.09945 | -0.23033 | -0.3271 | -0.12223 | -0.16331 | -0.17316 | -0.25465 | -0.19499 | -0.47843 |
| Ferulic acid | -0.2374 | -0.26612 | -0.02062 | -0.15949 | 0.07422 | -0.33172 | -0.15015 | 0.10692 | 0.25338 | 0.13569 | 0.43952 | 0.4465 | 0.34491 | 0.08949 |
| TPC | -0.09076 | -0.25642 | -0.01768 | -0.11256 | -0.13379 | -0.23218 | -0.01023 | -0.27368 | -0.06378 | 0.4673 | 0.59524 | 0.23616 | -0.02971 | 0.14222 |
| DPPH | -0.10191 | -0.27223 | -0.02467 | -0.1189 | -0.13982 | -0.23726 | -0.00097 | -0.2653 | -0.05314 | 0.47483 | 0.59774 | 0.25604 | -0.01214 | 0.15071 |
| Chlorogenic acid | 0.01554 | -0.08127 | 0.2166 | 0.10142 | -0.01373 | -0.07365 | 0.15289 | -0.25884 | -0.07891 | 0.44775 | 0.67235 | 0.32381 | 0.11053 | 0.31572 |
| ABTS | 0.05139 | -0.01036 | 0.23363 | 0.13178 | 0.01915 | -0.02745 | 0.13692 | -0.2769 | -0.08982 | 0.45186 | 0.67593 | 0.24888 | 0.01961 | 0.27775 |
| Isorhamnetin | 0.23459 | 0.27814 | 0.6402 | 0.49279 | 0.21678 | 0.22574 | 0.40831 | -0.19671 | -0.13076 | -0.09681 | 0.25308 | 0.40659 | 0.36689 | 0.58249 |
| Hesperidin | 0.39083 | 0.52914 | 0.82799 | 0.64309 | 0.36525 | 0.30976 | 0.32038 | -0.17264 | -0.31568 | -0.17044 | 0.31461 | 0.18108 | 0.42939 | 0.54434 |
| Ellagic acid | 0.36946 | 0.54224 | 0.87744 | 0.74655 | 0.38448 | 0.46949 | 0.57957 | -0.05413 | -0.11698 | 0.04128 | 0.47135 | 0.41432 | 0.55554 | 0.70651 |
| Vanillic acid | 0.0441 | -0.18145 | 0.14004 | -0.06327 | -0.12824 | -0.33216 | -0.15449 | -0.37139 | -0.32081 | -0.36206 | -0.11784 | 0.12207 | 0.09067 | 0.16088 |
| Phloretin | 0.3182 | 0.24012 | 0.5855 | 0.33796 | 0.11703 | -0.04092 | 0.01734 | -0.27183 | -0.45399 | -0.30236 | 0.17154 | 0.08309 | 0.39002 | 0.36709 |
| BChE inhibition | -0.04036 | 0.37572 | 0.43348 | 0.33828 | -0.01694 | 0.16992 | 43 | -0.14194 | -0.25372 | -0.21519 | 0.05189 | -0.10899 | 0.06539 | 161 |
| Gallic acid | 0.22922 | 0.19858 | 0.39914 | 0.24285 | -0.03646 | 0.0034 | 0.01145 | -0.25395 | -0.46703 | -0.44686 | -0.01905 | -0.09817 | 0.22506 | 0.40161 |
| 3,5-Dicaffeoylquin | 0.02931 | 0.16564 | 0.21667 | 0.10323 | 0.0286 | -0.05866 | -0.16599 | -0.58989 | -0.58813 | -0.67881 | -0.26578 | -0.3941 | -0.00151 | -0.00542 |
| TFC | 0.20265 | 0.58317 | 0.43554 | 0.44698 | 0.4885 | 0.41003 | 0.12147 | -0.2335 | -0.12668 | -0.16175 | -0.00043 | -0.24731 | -0.14355 | -0.12964 |
| Glucosidase inhibi | 1 | 0.53007 | 0.5041 | 0.46493 | 0.09351 | 0.35939 | 0.1672 | -0.07208 | -0.16016 | -0.14728 | 0.08042 | -0.11904 | 0.01019 | 0.18512 |
| Pelargonidin-3-glu | 0.53007 | 1 | 0.87749 | 0.92451 | 0.61601 | 0.87633 | 0.56405 | 0.0448 | -0.00557 | -0.01396 | 0.25172 | -0.05246 | 0.0194 | 0.4085 |
| Neochlorogenic aci | 0.5041 | 0.87749 | 1 | 0.96035 | 0.57495 | 0.78457 | 0.68035 | 0.00934 | -0.0436 | 0.04152 | 0.42809 | 0.23189 | 0.33796 | 0.65029 |
| CUPRAC | 0.46493 | 0.92451 | 0.96035 | 1 | 0.62609 | 0.92627 | 0.80254 | 0.11278 | 0.11891 | 0.17026 | 0.45378 | 0.26841 | 0.28003 | 0.64592 |
| Quercitrin | 0.09351 | 0.61601 | 0.57495 | 0.62609 | 1 | 0.61561 | 0.48644 | 0.24136 | 0.52015 | 0.25358 | 0.55022 | 0.4102 | 0.09721 | 362 |
| Syringic acid | 0.35939 | 0.87633 | 0.78457 | 0.92627 | 0.61561 | 1 | 0.85755 | 0.2325 | 0.31761 | 0.31338 | 0.42371 | 0.27095 | 0.15482 | 0.55013 |
| Quercetin | 0.1672 | 0.56405 | 0.68035 | 0.80254 | 0.48644 | 0.85755 | 1 | 0.29726 | 0.45926 | 0.52423 | 0.59604 | 0.64697 | 0.44545 | 0.72732 |
| Caffeic acid | -0.07208 | 0.0448 | 0.00934 | 0.11278 | 0.24136 | 0.2325 | 0.29726 | 1 | 0.47443 | 0.4134 | 0.14094 | 0.35342 | 0.40984 | 0.44343 |
| Catechin | -0.16016 | -0.00557 | -0.0436 | 0.11891 | 0.52015 | 0.31761 | 0.45926 | 0.47443 | 1 | 0.63499 | 0.61177 | 0.75818 | 0.10832 | 0.20582 |
| Phloridzin | -0.14728 | -0.01396 | 0.04152 | 0.17026 | 0.25358 | 0.31338 | 0.52423 | 0.4134 | 0.63499 | 1 | 0.73796 | 0.6183 | 0.05431 | 0.30827 |
| Cyanidin-3-glucosi | 0.08042 | 0.25172 | 0.42809 | 0.45378 | 0.55022 | 0.42371 | 0.59604 | 0.14094 | 0.61177 | 0.73796 | 1 | 0.75565 | 0.18934 | 0.51723 |
| Rutin | -0.11904 | -0.05246 | 0.23189 | 0.26841 | 0.4102 | 0.27095 | 0.64697 | 0.35342 | 0.75818 | 0.6183 | 0.75565 | 1 | 0.53586 | 0.56412 |
| p-Coumaric acid | 0.01019 | 0.0194 | 0.33796 | 0.28003 | 0.09721 | 0.15482 | 0.44545 | 0.40984 | 0.10832 | 0.05431 | 0.18934 | 0.53586 | 1 | 0.50209 |
| Kaempferol | 0.18512 | 0.4085 | 0.65029 | 0.64592 | 362 | 0.55013 | 0.72732 | 0.44343 | 0.20582 | 0.30827 | 0.51723 | 0.56412 | 0.50209 | 1 |

# **Table S4.** Pearson correlation coefficients between bioactive compounds and enzyme inhibition assays.

| | Cyanidin-3-glucosi | Rutin | p-Coumaric acid | Kaempferol | Catechin | Phloridzin | Quercitrin | Syringic acid | Quercetin | Delphindin-3,5- di | Hyperoside | Tyrosinase inhibit | Delphindin-3-galac | Malvidin-3-galacto | Kaempferol-3-gluco | Amylase inhibition | Caffeic acid | AChE inhibition | Epicatechin | Isoquercitrin | Procyanidin B2 | Pelargonidin-3-rut | Isorhamnetin | Hesperidin | Ellagic acid | Glucosidase inhibi | Neochlorogenic aci | Pelargonidin-3-glu | 4-Hydroxybenzoic | BChE inhibition | Gallic acid | 3,5-Dicaffeoylquin | Vanillic acid | Phloretin | Chlorogenic acid | Ferulic acid |
|---|---|---|---|---|---|---|---|---|---|---|---|---|---|---|---|---|---|---|---|---|---|---|---|---|---|---|---|---|---|---|---|---|---|---|---|---|
| **Cyanidin-3-glucosi** | 1 | 0.75565 | 0.18934 | 0.51723 | 0.61177 | 0.73796 | 0.55022 | 0.42371 | 0.59604 | -0.17437 | 0.07967 | 0.20872 | 0.10233 | 0.11925 | -0.18438 | -0.33649 | 0.14094 | -0.2041 | -0.0354 | -0.2143 | -0.36163 | -0.38443 | 0.25308 | 0.31461 | 0.47135 | 0.08042 | 0.42809 | 0.25172 | -0.18327 | 0.05189 | -0.01905 | -0.26578 | -0.11784 | 0.17154 | 0.67235 | 0.43952 |
| **Rutin** | 0.75565 | 1 | 0.53586 | 0.56412 | 0.75818 | 0.6183 | 0.4102 | 0.27095 | 0.64697 | -0.14009 | 0.08352 | 0.27987 | 0.17189 | 0.2454 | -0.28377 | -0.31168 | 0.35342 | -0.20241 | 0.22485 | -0.00205 | -0.19654 | -0.61236 | 0.40659 | 0.18108 | 0.41432 | -0.11904 | 0.23189 | -0.05246 | -0.28278 | -0.10899 | -0.09817 | -0.3941 | 0.12207 | 0.08309 | 0.32381 | 0.4465 |
| **p-Coumaric acid** | 0.18934 | 0.53586 | 1 | 0.50209 | 0.10832 | 0.05431 | 0.09721 | 0.15482 | 0.44545 | -0.42965 | -0.05517 | -0.07851 | -0.18268 | -0.13253 | -0.24935 | -0.33074 | 0.40984 | -0.09042 | -0.3161 | 0.50633 | -0.05855 | -0.23205 | 0.36689 | 0.42939 | 0.55554 | 0.01019 | 0.33796 | 0.0194 | -0.14157 | 0.06539 | 0.22506 | -0.00151 | 0.09067 | 0.39002 | 0.11053 | 0.34491 |
| **Kaempferol** | 0.51723 | 0.56412 | 0.50209 | 1 | 0.20582 | 0.30827 | 362 | 0.55013 | 0.72732 | -0.35763 | 0.1262 | 0.13405 | -0.00976 | -0.00169 | -0.28796 | -0.68899 | 0.44343 | 0.32666 | -0.38964 | -0.23309 | -0.45801 | -0.34546 | 0.58249 | 0.54434 | 0.70651 | 0.18512 | 0.65029 | 0.4085 | 0.34871 | 161 | 0.40161 | -0.00542 | 0.16088 | 0.36709 | 0.31572 | 0.08949 |
| **Catechin** | 0.61177 | 0.75818 | 0.10832 | 0.20582 | 1 | 0.63499 | 0.52015 | 0.31761 | 0.45926 | 0.43487 | 0.28667 | 0.62645 | 0.59758 | 0.62532 | -0.01351 | -0.08005 | 0.47443 | -0.14678 | 0.44182 | -0.10036 | -0.34272 | -0.67759 | -0.13076 | -0.31568 | -0.11698 | -0.16016 | -0.0436 | -0.00557 | -0.25687 | -0.25372 | -0.46703 | -0.58813 | -0.32081 | -0.45399 | -0.07891 | 0.25338 |
| **Phloridzin** | 0.73796 | 0.6183 | 0.05431 | 0.30827 | 0.63499 | 1 | 0.25358 | 0.31338 | 0.52423 | 0.27069 | 0.17702 | 0.53308 | 0.48092 | 0.52177 | -0.18589 | -0.16503 | 0.4134 | 0.25844 | -0.00102 | -0.24134 | -0.30965 | -0.6687 | -0.09681 | -0.17044 | 0.04128 | -0.14728 | 0.04152 | -0.01396 | -0.26796 | -0.21519 | -0.44686 | -0.67881 | -0.36206 | -0.30236 | 0.44775 | 0.13569 |
| **Quercitrin** | 0.55022 | 0.4102 | 0.09721 | 362 | 0.52015 | 0.25358 | 1 | 0.61561 | 0.48644 | -0.04654 | -0.03237 | 0.22894 | 0.14733 | 0.08422 | 0.00746 | -0.31228 | 0.24136 | -0.19482 | 0.05625 | -0.09204 | -0.59668 | -0.14425 | 0.21678 | 0.36525 | 0.38448 | 0.09351 | 0.57495 | 0.61601 | -0.0626 | -0.01694 | -0.03646 | 0.0286 | -0.12824 | 0.11703 | -0.01373 | 0.07422 |
| **Syringic acid** | 0.42371 | 0.27095 | 0.15482 | 0.55013 | 0.31761 | 0.31338 | 0.61561 | 1 | 0.85755 | 0.13588 | 0.48598 | 0.58909 | 0.46596 | 0.38682 | 0.31184 | -0.44214 | 0.2325 | 0.07107 | -0.39133 | -0.17526 | -0.98834 | -0.47065 | 0.22574 | 0.30976 | 0.46949 | 0.35939 | 0.78457 | 0.87633 | -0.18668 | 0.16992 | 0.0034 | -0.05866 | -0.33216 | -0.04092 | -0.07365 | -0.33172 |
| **Quercetin** | 0.59604 | 0.64697 | 0.44545 | 0.72732 | 0.45926 | 0.52423 | 0.48644 | 0.85755 | 1 | 0.0111 | 0.44061 | 0.58803 | 0.43552 | 0.4284 | 0.1218 | -0.49938 | 0.29726 | 0.04033 | -0.31825 | -0.1222 | -0.80305 | -0.71794 | 0.40831 | 0.32038 | 0.57957 | 0.1672 | 0.68035 | 0.56405 | -0.30975 | 43 | 0.01145 | -0.16599 | -0.15449 | 0.01734 | 0.15289 | -0.15015 |
| **Delphindin-3,5- di** | -0.17437 | -0.14009 | -0.42965 | -0.35763 | 0.43487 | 0.27069 | -0.04654 | 0.13588 | 0.0111 | 1 | 0.51409 | 0.81456 | 0.90396 | 0.88908 | 0.46238 | 0.28089 | 0.26545 | 0.22837 | 0.19566 | -0.16546 | -0.27345 | -0.55562 | -0.76843 | -0.89218 | -0.7949 | -0.24688 | -0.49722 | -0.1116 | -0.1509 | -0.40989 | -0.62031 | -0.37717 | -0.71638 | -0.98986 | -0.51553 | -0.46801 |
| **Hyperoside** | 0.07967 | 0.08352 | -0.05517 | 0.1262 | 0.28667 | 0.17702 | -0.03237 | 0.48598 | 0.44061 | 0.51409 | 1 | 0.66931 | 0.64657 | 0.62569 | 0.47225 | -0.05759 | 0.09154 | 0.03256 | -0.15259 | -0.14581 | -0.53498 | -0.5567 | -0.2185 | -316 | -0.13992 | 0.53506 | 0.09066 | 0.26121 | -0.19436 | -0.42985 | -0.15869 | -0.1436 | -0.45125 | -0.48326 | -0.22047 | -0.57344 |
| **Tyrosinase inhibit** | 0.20872 | 0.27987 | -0.07851 | 0.13405 | 0.62645 | 0.53308 | 0.22894 | 0.58909 | 0.58803 | 0.81456 | 0.66931 | 1 | 0.98419 | 0.97302 | 0.43597 | -0.05708 | 0.38858 | 0.20602 | -0.01593 | -0.2034 | -0.66747 | -0.87963 | -0.38187 | -0.54594 | -0.31192 | -0.11849 | -0.02627 | 0.2081 | -0.3081 | -0.31915 | -0.49769 | -0.40923 | -0.65967 | -0.79471 | -0.31839 | -0.45568 |
| **Delphindin-3-galac** | 0.10233 | 0.17189 | -0.18268 | -0.00976 | 0.59758 | 0.48092 | 0.14733 | 0.46596 | 0.43552 | 0.90396 | 0.64657 | 0.98419 | 1 | 0.98966 | 0.45543 | 0.04502 | 0.36953 | 0.22188 | 0.05284 | -0.19761 | -0.56497 | -0.82336 | -0.51461 | -0.67861 | -0.47445 | -0.17128 | -0.18013 | 0.10501 | -0.27581 | -366 | -0.55858 | -0.42126 | -0.70014 | -0.88863 | -0.38666 | -0.47273 |
| **Malvidin-3-galacto** | 0.11925 | 0.2454 | -0.13253 | -0.00169 | 0.62532 | 0.52177 | 0.08422 | 0.38682 | 0.4284 | 0.88908 | 0.62569 | 0.97302 | 0.98966 | 1 | 0.40733 | 0.06173 | 0.37765 | 0.21601 | 0.09375 | -0.1848 | -0.47913 | -0.87145 | -0.49677 | -0.71034 | -0.48782 | -0.2368 | -0.25332 | -0.01389 | -0.29935 | -0.41308 | -0.5679 | -0.45337 | -0.65494 | -0.89393 | -0.34019 | -0.4246 |
| **Kaempferol-3-gluco** | -0.18438 | -0.28377 | -0.24935 | -0.28796 | -0.01351 | -0.18589 | 0.00746 | 0.31184 | 0.1218 | 0.46238 | 0.47225 | 0.43597 | 0.45543 | 0.40733 | 1 | 0.57668 | -0.32721 | -0.27905 | -0.30919 | -0.35473 | -0.37897 | -0.21024 | -0.32258 | -0.28519 | -0.25486 | 0.05559 | 0.01055 | 0.25985 | -0.34327 | -0.06654 | 0.19537 | 0.28397 | -0.41366 | -0.40859 | -0.30329 | -0.47727 |
| **Amylase inhibition** | -0.33649 | -0.31168 | -0.33074 | -0.68899 | -0.08005 | -0.16503 | -0.31228 | -0.44214 | -0.49938 | 0.28089 | -0.05759 | -0.05708 | 0.04502 | 0.06173 | 0.57668 | 1 | -0.30062 | -0.23347 | 0.09619 | -0.29684 | 0.37925 | 0.15886 | -0.40727 | -0.44862 | -0.53405 | -0.18197 | -0.53648 | -0.39657 | -0.26693 | -171 | 73 | -0.04148 | -0.08843 | -0.30312 | -0.19639 | -0.03749 |
| **Caffeic acid** | 0.14094 | 0.35342 | 0.40984 | 0.44343 | 0.47443 | 0.4134 | 0.24136 | 0.2325 | 0.29726 | 0.26545 | 0.09154 | 0.38858 | 0.36953 | 0.37765 | -0.32721 | -0.30062 | 1 | 0.6168 | -0.09346 | 0.06129 | -0.24905 | -0.4073 | -0.19671 | -0.17264 | -0.05413 | -0.07208 | 0.00934 | 0.0448 | 0.34284 | -0.14194 | -0.25395 | -0.58989 | -0.37139 | -0.27183 | -0.25884 | 0.10692 |
| **AChE inhibition** | -0.2041 | -0.20241 | -0.09042 | 0.32666 | -0.14678 | 0.25844 | -0.19482 | 0.07107 | 0.04033 | 0.22837 | 0.03256 | 0.20602 | 0.22188 | 0.21601 | -0.27905 | -0.23347 | 0.6168 | 1 | -0.30911 | -0.29584 | -0.10126 | -0.14861 | -0.15811 | -0.18704 | -0.15695 | -0.03871 | -0.0803 | 0.00798 | 0.57497 | -0.08983 | -0.08687 | -0.35896 | -0.16924 | -0.22533 | -0.11625 | -0.33498 |
| **Epicatechin** | -0.0354 | 0.22485 | -0.3161 | -0.38964 | 0.44182 | -0.00102 | 0.05625 | -0.39133 | -0.31825 | 0.19566 | -0.15259 | -0.01593 | 0.05284 | 0.09375 | -0.30919 | 0.09619 | -0.09346 | -0.30911 | 1 | 0.14569 | 0.35669 | -0.00302 | -0.01137 | -0.36604 | -0.38671 | -0.24517 | -0.46988 | -0.43819 | -0.16637 | -0.20139 | -0.54388 | -0.32214 | 0.31406 | -0.24422 | -0.28264 | 0.22835 |
| **Isoquercitrin** | -0.2143 | -0.00205 | 0.50633 | -0.23309 | -0.10036 | -0.24134 | -0.09204 | -0.17526 | -0.1222 | -0.16546 | -0.14581 | -0.2034 | -0.19761 | -0.1848 | -0.35473 | -0.29684 | 0.06129 | -0.29584 | 0.14569 | 1 | 0.19428 | 0.13288 | -0.07392 | 0.07827 | 0.04626 | -0.03085 | -0.05515 | -0.13256 | -0.29164 | 0.01725 | -0.36436 | 0.05538 | -0.05125 | 0.14384 | -0.16327 | 0.22424 |
| **Procyanidin B2** | -0.36163 | -0.19654 | -0.05855 | -0.45801 | -0.34272 | -0.30965 | -0.59668 | -0.98834 | -0.80305 | -0.27345 | -0.53498 | -0.66747 | -0.56497 | -0.47913 | -0.37897 | 0.37925 | -0.24905 | -0.10126 | 0.35669 | 0.19428 | 1 | 0.49202 | -0.09459 | -0.17905 | -0.33239 | -0.33306 | -0.69651 | -0.85818 | 0.18326 | -0.12396 | 0.07833 | 0.09239 | 0.4305 | 0.17378 | 0.16334 | 0.39807 |
| **Pelargonidin-3-rut** | -0.38443 | -0.61236 | -0.23205 | -0.34546 | -0.67759 | -0.6687 | -0.14425 | -0.47065 | -0.71794 | -0.55562 | -0.5567 | -0.87963 | -0.82336 | -0.87145 | -0.21024 | 0.15886 | -0.4073 | -0.14861 | -0.00302 | 0.13288 | 0.49202 | 1 | 0.10848 | 0.38828 | 0.08635 | 0.22962 | 0.0163 | 0.01074 | 0.38724 | 0.35326 | 0.39473 | 0.43921 | 0.40983 | 0.58957 | 0.05342 | 0.2383 |
| **Isorhamnetin** | 0.25308 | 0.40659 | 0.36689 | 0.58249 | -0.13076 | -0.09681 | 0.21678 | 0.22574 | 0.40831 | -0.76843 | -0.2185 | -0.38187 | -0.51461 | -0.49677 | -0.32258 | -0.40727 | -0.19671 | -0.15811 | -0.01137 | -0.07392 | -0.09459 | 0.10848 | 1 | 0.80732 | 0.84809 | 0.23459 | 0.6402 | 0.27814 | -0.02291 | 0.31769 | 0.47663 | 0.20855 | 0.81285 | 765 | 0.28561 | 0.16443 |
| **Hesperidin** | 0.31461 | 0.18108 | 0.42939 | 0.54434 | -0.31568 | -0.17044 | 0.36525 | 0.30976 | 0.32038 | -0.89218 | -316 | -0.54594 | -0.67861 | -0.71034 | -0.28519 | -0.44862 | -0.17264 | -0.18704 | -0.36604 | 0.07827 | -0.17905 | 0.38828 | 0.80732 | 1 | 0.94754 | 0.39083 | 0.82799 | 0.52914 | 0.09103 | 0.45748 | 0.60232 | 0.39443 | 0.52551 | 0.93382 | 0.42924 | 0.2654 |
| **Ellagic acid** | 0.47135 | 0.41432 | 0.55554 | 0.70651 | -0.11698 | 0.04128 | 0.38448 | 0.46949 | 0.57957 | -0.7949 | -0.13992 | -0.31192 | -0.47445 | -0.48782 | -0.25486 | -0.53405 | -0.05413 | -0.15695 | -0.38671 | 0.04626 | -0.33239 | 0.08635 | 0.84809 | 0.94754 | 1 | 0.36946 | 0.87744 | 0.54224 | -0.03239 | 0.40864 | 0.52505 | 0.24351 | 0.45063 | 0.82408 | 0.46179 | 0.24407 |
| **Glucosidase inhibi** | 0.08042 | -0.11904 | 0.01019 | 0.18512 | -0.16016 | -0.14728 | 0.09351 | 0.35939 | 0.1672 | -0.24688 | 0.53506 | -0.11849 | -0.17128 | -0.2368 | 0.05559 | -0.18197 | -0.07208 | -0.03871 | -0.24517 | -0.03085 | -0.33306 | 0.22962 | 0.23459 | 0.39083 | 0.36946 | 1 | 0.5041 | 0.53007 | 0.08211 | -0.04036 | 0.22922 | 0.02931 | 0.0441 | 0.3182 | 0.01554 | -0.2374 |
| **Neochlorogenic aci** | 0.42809 | 0.23189 | 0.33796 | 0.65029 | -0.0436 | 0.04152 | 0.57495 | 0.78457 | 0.68035 | -0.49722 | 0.09066 | -0.02627 | -0.18013 | -0.25332 | 0.01055 | -0.53648 | 0.00934 | -0.0803 | -0.46988 | -0.05515 | -0.69651 | 0.0163 | 0.6402 | 0.82799 | 0.87744 | 0.5041 | 1 | 0.87749 | -0.03231 | 0.43348 | 0.39914 | 0.21667 | 0.14004 | 0.5855 | 0.2166 | -0.02062 |
| **Pelargonidin-3-glu** | 0.25172 | -0.05246 | 0.0194 | 0.4085 | -0.00557 | -0.01396 | 0.61601 | 0.87633 | 0.56405 | -0.1116 | 0.26121 | 0.2081 | 0.10501 | -0.01389 | 0.25985 | -0.39657 | 0.0448 | 0.00798 | -0.43819 | -0.13256 | -0.85818 | 0.01074 | 0.27814 | 0.52914 | 0.54224 | 0.53007 | 0.87749 | 1 | 0.00262 | 0.37572 | 0.19858 | 0.16564 | -0.18145 | 0.24012 | -0.08127 | -0.26612 |
| **4-Hydroxybenzoic** | -0.18327 | -0.28278 | -0.14157 | 0.34871 | -0.25687 | -0.26796 | -0.0626 | -0.18668 | -0.30975 | -0.1509 | -0.19436 | -0.3081 | -0.27581 | -0.29935 | -0.34327 | -0.26693 | 0.34284 | 0.57497 | -0.16637 | -0.29164 | 0.18326 | 0.38724 | -0.02291 | 0.09103 | -0.03239 | 0.08211 | -0.03231 | 0.00262 | 1 | 0.11978 | 0.40082 | 0.15808 | 0.11041 | 0.16786 | -0.02579 | 0.05872 |
| **BChE inhibition** | 0.05189 | -0.10899 | 0.06539 | 161 | -0.25372 | -0.21519 | -0.01694 | 0.16992 | 43 | -0.40989 | -0.42985 | -0.31915 | -366 | -0.41308 | -0.06654 | -171 | -0.14194 | -0.08983 | -0.20139 | 0.01725 | -0.12396 | 0.35326 | 0.31769 | 0.45748 | 0.40864 | -0.04036 | 0.43348 | 0.37572 | 0.11978 | 1 | 0.31473 | 0.07524 | 0.20904 | 0.45869 | 0.11519 | 0.45407 |
| **Gallic acid** | -0.01905 | -0.09817 | 0.22506 | 0.40161 | -0.46703 | -0.44686 | -0.03646 | 0.0034 | 0.01145 | -0.62031 | -0.15869 | -0.49769 | -0.55858 | -0.5679 | 0.19537 | 73 | -0.25395 | -0.08687 | -0.54388 | -0.36436 | 0.07833 | 0.39473 | 0.47663 | 0.60232 | 0.52505 | 0.22922 | 0.39914 | 0.19858 | 0.40082 | 0.31473 | 1 | 0.6223 | 0.4102 | 0.63853 | 0.2825 | 0.10633 |
| **3,5-Dicaffeoylquin** | -0.26578 | -0.3941 | -0.00151 | -0.00542 | -0.58813 | -0.67881 | 0.0286 | -0.05866 | -0.16599 | -0.37717 | -0.1436 | -0.40923 | -0.42126 | -0.45337 | 0.28397 | -0.04148 | -0.58989 | -0.35896 | -0.32214 | 0.05538 | 0.09239 | 0.43921 | 0.20855 | 0.39443 | 0.24351 | 0.02931 | 0.21667 | 0.16564 | 0.15808 | 0.07524 | 0.6223 | 1 | 0.23652 | 0.40809 | 0.09548 | -0.20454 |
| **Vanillic acid** | -0.11784 | 0.12207 | 0.09067 | 0.16088 | -0.32081 | -0.36206 | -0.12824 | -0.33216 | -0.15449 | -0.71638 | -0.45125 | -0.65967 | -0.70014 | -0.65494 | -0.41366 | -0.08843 | -0.37139 | -0.16924 | 0.31406 | -0.05125 | 0.4305 | 0.40983 | 0.81285 | 0.52551 | 0.45063 | 0.0441 | 0.14004 | -0.18145 | 0.11041 | 0.20904 | 0.4102 | 0.23652 | 1 | 0.67448 | 0.15093 | 0.21875 |
| **Phloretin** | 0.17154 | 0.08309 | 0.39002 | 0.36709 | -0.45399 | -0.30236 | 0.11703 | -0.04092 | 0.01734 | -0.98986 | -0.48326 | -0.79471 | -0.88863 | -0.89393 | -0.40859 | -0.30312 | -0.27183 | -0.22533 | -0.24422 | 0.14384 | 0.17378 | 0.58957 | 765 | 0.93382 | 0.82408 | 0.3182 | 0.5855 | 0.24012 | 0.16786 | 0.45869 | 0.63853 | 0.40809 | 0.67448 | 1 | 0.47593 | 419 |
| **Chlorogenic acid** | 0.67235 | 0.32381 | 0.11053 | 0.31572 | -0.07891 | 0.44775 | -0.01373 | -0.07365 | 0.15289 | -0.51553 | -0.22047 | -0.31839 | -0.38666 | -0.34019 | -0.30329 | -0.19639 | -0.25884 | -0.11625 | -0.28264 | -0.16327 | 0.16334 | 0.05342 | 0.28561 | 0.42924 | 0.46179 | 0.01554 | 0.2166 | -0.08127 | -0.02579 | 0.11519 | 0.2825 | 0.09548 | 0.15093 | 0.47593 | 1 | 0.42271 |
| **Ferulic acid** | 0.43952 | 0.4465 | 0.34491 | 0.08949 | 0.25338 | 0.13569 | 0.07422 | -0.33172 | -0.15015 | -0.46801 | -0.57344 | -0.45568 | -0.47273 | -0.4246 | -0.47727 | -0.03749 | 0.10692 | -0.33498 | 0.22835 | 0.22424 | 0.39807 | 0.2383 | 0.16443 | 0.2654 | 0.24407 | -0.2374 | -0.02062 | -0.26612 | 0.05872 | 0.45407 | 0.10633 | -0.20454 | 0.21875 | 419 | 0.42271 | 1 |

# **Table S5.** *P*-value between bioactive compounds and antioxidant assays.

| | Kaempferol | p-Coumaric acid | Rutin | Cyanidin-3-glucosi | Phloridzin | Catechin | Caffeic acid | Quercetin | Syringic acid | Quercitrin | CUPRAC | Neochlorogenic aci | Pelargonidin-3-glu | Glucosidase inhibi | TFC | 3,5-Dicaffeoylquin | Gallic acid | BChE inhibition | Phloretin | Vanillic acid | Ellagic acid | Hesperidin |
|---|---|---|---|---|---|---|---|---|---|---|---|---|---|---|---|---|---|---|---|---|---|---|
| **Kaempferol** | NA | 0.096234 | 0.056063 | 0.085045 | 0.32963 | 0.52103 | 0.14877 | 0.0073489 | 0.063848 | 0.24756 | 0.023268 | 0.02205 | 0.18738 | 0.56461 | 688 | 0.98667 | 0.19566 | 0.61716 | 0.24047 | 0.61743 | 0.010212 | 0.067284 |
| **p-Coumaric acid** | 0.096234 | NA | 0.072532 | 0.55562 | 0.86688 | 0.73757 | 0.18579 | 0.14671 | 0.63091 | 0.76377 | 0.37802 | 0.28263 | 0.95227 | 0.97492 | 0.65626 | 0.99629 | 0.48189 | 0.83999 | 0.21008 | 0.77929 | 0.060757 | 0.16362 |
| **Rutin** | 0.056063 | 0.072532 | NA | 0.0044745 | 0.032112 | 0.0042673 | 0.25976 | 0.022971 | 0.39431 | 0.18536 | 0.39894 | 0.46831 | 0.87138 | 0.71252 | 0.43837 | 0.20493 | 0.76148 | 0.73599 | 0.79739 | 0.70547 | 0.18055 | 0.57329 |
| **Cyanidin-3-glucosi** | 0.085045 | 0.55562 | 0.0044745 | NA | 0.0061434 | 0.034514 | 0.66217 | 0.04082 | 0.16988 | 0.063797 | 0.13841 | 0.16503 | 0.42998 | 0.80379 | 0.99894 | 0.40375 | 0.95313 | 0.87275 | 0.59398 | 0.7153 | 0.1219 | 0.31927 |
| **Phloridzin** | 0.32963 | 0.86688 | 0.032112 | 0.0061434 | NA | 0.026525 | 0.18161 | 0.080186 | 0.32125 | 0.42645 | 0.59677 | 0.89806 | 0.96564 | 0.64784 | 0.6155 | 0.015216 | 0.14529 | 0.50179 | 0.33948 | 0.24747 | 0.89864 | 0.59639 |
| **Catechin** | 0.52103 | 0.73757 | 0.0042673 | 0.034514 | 0.026525 | NA | 0.11914 | 0.13311 | 0.31442 | 0.082995 | 0.71282 | 0.89297 | 0.98628 | 0.61903 | 0.69482 | 0.044283 | 0.12583 | 0.42619 | 0.1382 | 0.30929 | 0.7173 | 0.31753 |
| **Caffeic acid** | 0.14877 | 0.18579 | 0.25976 | 0.66217 | 0.18161 | 0.11914 | NA | 0.34809 | 0.46712 | 0.44982 | 0.72712 | 0.97701 | 0.89005 | 0.82385 | 0.46514 | 0.043498 | 0.42576 | 0.6599 | 0.39271 | 0.23459 | 0.86731 | 0.59157 |
| **Quercetin** | 0.0073489 | 0.14671 | 0.022971 | 0.04082 | 0.080186 | 0.13311 | 0.34809 | NA | 0.00036184 | 0.1088 | 0.0016791 | 0.014897 | 0.056098 | 0.6035 | 0.70687 | 0.60615 | 0.97182 | 0.89445 | 0.95734 | 0.63164 | 0.04826 | 0.30998 |
| **Syringic acid** | 0.063848 | 0.63091 | 0.39431 | 0.16988 | 0.32125 | 0.31442 | 0.46712 | 0.00036184 | NA | 0.033087 | 1.5148e-05 | 0.0025125 | 0.00018443 | 0.25123 | 0.18557 | 0.85629 | 0.99163 | 0.59752 | 0.89953 | 0.2915 | 0.12358 | 0.32718 |
| **Quercitrin** | 0.24756 | 0.76377 | 0.18536 | 0.063797 | 0.42645 | 0.082995 | 0.44982 | 0.1088 | 0.033087 | NA | 0.029407 | 0.050509 | 0.03294 | 0.77254 | 0.10708 | 0.9297 | 0.91044 | 0.95832 | 0.71718 | 0.69123 | 0.21719 | 0.24301 |
| **CUPRAC** | 0.023268 | 0.37802 | 0.39894 | 0.13841 | 0.59677 | 0.71282 | 0.72712 | 0.0016791 | 1.5148e-05 | 0.029407 | NA | 7.2229e-07 | 1.6989e-05 | 0.12777 | 0.14517 | 0.74953 | 0.44693 | 0.28215 | 0.28263 | 0.84514 | 0.0052832 | 0.024081 |
| **Neochlorogenic aci** | 0.02205 | 0.28263 | 0.46831 | 0.16503 | 0.89806 | 0.89297 | 0.97701 | 0.014897 | 0.0025125 | 0.050509 | 7.2229e-07 | NA | 0.00017634 | 0.094697 | 157 | 0.49878 | 0.19867 | 0.15919 | 0.045483 | 0.66423 | 0.0001767 | 0.00088173 |
| **Pelargonidin-3-glu** | 0.18738 | 0.95227 | 0.87138 | 0.42998 | 0.96564 | 0.98628 | 0.89005 | 0.056098 | 0.00018443 | 0.03294 | 1.6989e-05 | 0.00017634 | NA | 0.076278 | 0.04656 | 0.60691 | 0.5361 | 0.22874 | 0.45221 | 0.57249 | 0.068555 | 0.076892 |
| **Glucosidase inhibi** | 0.56461 | 0.97492 | 0.71252 | 0.80379 | 0.64784 | 0.61903 | 0.82385 | 0.6035 | 0.25123 | 0.77254 | 0.12777 | 0.094697 | 0.076278 | NA | 0.52761 | 0.92794 | 0.4736 | 0.90088 | 0.31346 | 0.89177 | 0.23722 | 0.20905 |
| **TFC** | 688 | 0.65626 | 0.43837 | 0.99894 | 0.6155 | 0.69482 | 0.46514 | 0.70687 | 0.18557 | 0.10708 | 0.14517 | 157 | 0.04656 | 0.52761 | NA | 0.34163 | 0.32911 | 0.7398 | 0.68628 | 0.70321 | 0.53606 | 0.35729 |
| **3,5-Dicaffeoylquin** | 0.98667 | 0.99629 | 0.20493 | 0.40375 | 0.015216 | 0.044283 | 0.043498 | 0.60615 | 0.85629 | 0.9297 | 0.74953 | 0.49878 | 0.60691 | 0.92794 | 0.34163 | NA | 0.030704 | 0.81622 | 0.18786 | 0.45923 | 0.44566 | 0.20452 |
| **Gallic acid** | 0.19566 | 0.48189 | 0.76148 | 0.95313 | 0.14529 | 0.12583 | 0.42576 | 0.97182 | 0.99163 | 0.91044 | 0.44693 | 0.19867 | 0.5361 | 0.4736 | 0.32911 | 0.030704 | NA | 0.31906 | 0.025434 | 0.18536 | 0.079631 | 0.038212 |
| **BChE inhibition** | 0.61716 | 0.83999 | 0.73599 | 0.87275 | 0.50179 | 0.42619 | 0.6599 | 0.89445 | 0.59752 | 0.95832 | 0.28215 | 0.15919 | 0.22874 | 0.90088 | 0.7398 | 0.81622 | 0.31906 | NA | 0.13365 | 0.51438 | 0.1872 | 0.13481 |
| **Phloretin** | 0.24047 | 0.21008 | 0.79739 | 0.59398 | 0.33948 | 0.1382 | 0.39271 | 0.95734 | 0.89953 | 0.71718 | 0.28263 | 0.045483 | 0.45221 | 0.31346 | 0.68628 | 0.18786 | 0.025434 | 0.13365 | NA | 0.016137 | 0.00097963 | 8.94e-06 |
| **Vanillic acid** | 0.61743 | 0.77929 | 0.70547 | 0.7153 | 0.24747 | 0.30929 | 0.23459 | 0.63164 | 0.2915 | 0.69123 | 0.84514 | 0.66423 | 0.57249 | 0.89177 | 0.70321 | 0.45923 | 0.18536 | 0.51438 | 0.016137 | NA | 0.14151 | 0.079321 |
| **Ellagic acid** | 0.010212 | 0.060757 | 0.18055 | 0.1219 | 0.89864 | 0.7173 | 0.86731 | 0.04826 | 0.12358 | 0.21719 | 0.0052832 | 0.0001767 | 0.068555 | 0.23722 | 0.53606 | 0.44566 | 0.079631 | 0.1872 | 0.00097963 | 0.14151 | NA | 2.8649e-06 |
| **Hesperidin** | 0.067284 | 0.16362 | 0.57329 | 0.31927 | 0.59639 | 0.31753 | 0.59157 | 0.30998 | 0.32718 | 0.24301 | 0.024081 | 0.00088173 | 0.076892 | 0.20905 | 0.35729 | 0.20452 | 0.038212 | 0.13481 | 8.94e-06 | 0.079321 | 2.8649e-06 | NA |
| **Isorhamnetin** | 0.046878 | 0.24075 | 0.18964 | 0.4274 | 0.76471 | 0.68543 | 0.54004 | 0.1876 | 0.48051 | 0.49857 | 0.10358 | 0.024933 | 0.38138 | 0.46301 | 0.87418 | 0.5154 | 0.1172 | 0.31429 | 0.0037451 | 0.0013082 | 0.00049086 | 0.0014983 |
| **ABTS** | 0.38208 | 0.95177 | 0.43537 | 0.015823 | 0.14029 | 0.78132 | 0.38359 | 0.67132 | 0.93253 | 0.9529 | 0.68309 | 0.46489 | 0.9745 | 0.87396 | 0.82554 | 0.74583 | 0.40951 | 0.68556 | 0.15785 | 0.83132 | 0.17048 | 0.19123 |
| **Chlorogenic acid** | 0.31746 | 0.73239 | 0.30453 | 0.016603 | 0.14439 | 0.8074 | 0.41659 | 0.63523 | 0.82006 | 0.96622 | 0.7538 | 0.49892 | 0.80175 | 0.96177 | 0.67998 | 0.76787 | 0.37365 | 0.72149 | 0.11782 | 0.63963 | 0.13071 | 0.16378 |
| **DPPH** | 0.64012 | 0.97013 | 0.42183 | 0.040105 | 0.11879 | 0.86971 | 0.40464 | 0.99761 | 0.45779 | 0.66473 | 0.71285 | 0.93933 | 392 | 0.75265 | 0.48374 | 0.91831 | 0.60629 | 0.99042 | 0.34428 | 0.84326 | 0.47545 | 0.52303 |
| **TPC** | 0.65927 | 0.92698 | 0.45994 | 0.041162 | 0.12558 | 0.84389 | 0.38937 | 0.97483 | 0.46774 | 0.67849 | 0.72763 | 0.9565 | 0.42112 | 0.77909 | 0.51395 | 0.89868 | 0.59932 | 0.96871 | 0.34 | 0.86396 | 0.47996 | 0.51496 |

| | Isorhamnetin | ABTS | Chlorogenic acid | DPPH | TPC | Ferulic acid | FRAP | Isoquercitrin | Epicatechin | AChE inhibition | Metal chelating | 4-Hydroxybenzoic | Amylase inhibition | Phosphomolybdenum | Kaempferol-3-gluco | Pelargonidin-3-rut | Procyanidin B2 | Malvidin-3-galacto | Delphindin-3-galac | Tyrosinase inhibit | Hyperoside | Delphindin-3,5- di |
|---|---|---|---|---|---|---|---|---|---|---|---|---|---|---|---|---|---|---|---|---|---|---|
| **Kaempferol** | 0.046878 | 0.38208 | 0.31746 | 0.64012 | 0.65927 | 0.78211 | 0.11563 | 0.46595 | 0.21056 | 0.30005 | 0.70188 | 0.26661 | 0.013205 | 0.067053 | 0.36408 | 0.2714 | 0.1343 | 0.99583 | 0.97598 | 0.67789 | 0.69593 | 0.25373 |
| **p-Coumaric acid** | 0.24075 | 0.95177 | 0.73239 | 0.97013 | 0.92698 | 0.27222 | 0.54366 | 0.093009 | 0.31684 | 0.77989 | 0.3215 | 0.66074 | 0.29369 | 0.40932 | 0.43448 | 0.46799 | 0.85658 | 0.68138 | 0.56984 | 0.80837 | 0.86478 | 0.16333 |
| **Rutin** | 0.18964 | 0.43537 | 0.30453 | 0.42183 | 0.45994 | 0.14565 | 0.42443 | 0.99495 | 0.4823 | 0.5281 | 0.26126 | 0.37315 | 0.32404 | 0.33102 | 0.3714 | 0.034291 | 0.54039 | 0.44203 | 0.59321 | 0.37831 | 0.79636 | 0.6641 |
| **Cyanidin-3-glucosi** | 0.4274 | 0.015823 | 0.016603 | 0.040105 | 0.041162 | 0.15282 | 0.59044 | 0.50361 | 0.91304 | 0.52459 | 0.55805 | 0.56858 | 0.28486 | 0.48321 | 0.56621 | 0.21727 | 0.24807 | 0.71202 | 0.75164 | 0.51504 | 0.80558 | 0.58782 |
| **Phloridzin** | 0.76471 | 0.14029 | 0.14439 | 0.11879 | 0.12558 | 0.67413 | 0.61206 | 0.44985 | 0.99749 | 0.41733 | 0.62483 | 0.39977 | 0.60826 | 0.59074 | 0.56297 | 0.017429 | 0.32736 | 0.081869 | 0.11348 | 0.074317 | 0.58205 | 0.39479 |
| **Catechin** | 0.68543 | 0.78132 | 0.8074 | 0.86971 | 0.84389 | 0.42684 | 0.70512 | 0.75632 | 0.15043 | 0.64896 | 0.81249 | 0.42026 | 0.80468 | 0.72682 | 0.96676 | 0.015471 | 0.27548 | 0.029669 | 0.040169 | 0.029288 | 0.36633 | 0.15771 |
| **Caffeic acid** | 0.54004 | 0.38359 | 0.41659 | 0.40464 | 0.38937 | 0.74086 | 0.29936 | 0.84991 | 0.77267 | 0.032652 | 0.3563 | 0.27529 | 0.34239 | 0.31554 | 0.29918 | 0.1888 | 0.43504 | 0.22617 | 0.23712 | 0.21191 | 0.77722 | 0.40435 |
| **Quercetin** | 0.1876 | 0.67132 | 0.63523 | 0.99761 | 0.97483 | 0.64138 | 0.47141 | 0.70517 | 0.31339 | 0.90096 | 0.62986 | 0.3272 | 0.098335 | 0.36279 | 0.70611 | 0.0085528 | 0.0016592 | 0.16469 | 0.15702 | 0.044332 | 0.15169 | 0.97269 |
| **Syringic acid** | 0.48051 | 0.93253 | 0.82006 | 0.45779 | 0.46774 | 0.29217 | 0.75845 | 0.58587 | 0.20841 | 0.82627 | 0.75645 | 0.56129 | 0.1501 | 0.67299 | 0.32378 | 0.12253 | 1.6631e-09 | 0.21418 | 0.12682 | 0.043855 | 0.10918 | 0.67371 |
| **Quercitrin** | 0.49857 | 0.9529 | 0.96622 | 0.66473 | 0.67849 | 0.81869 | 0.89628 | 0.77603 | 0.86215 | 0.54401 | 0.80997 | 0.84675 | 0.32306 | 0.81741 | 0.98164 | 0.65467 | 0.040551 | 0.79468 | 0.64773 | 0.47416 | 0.92046 | 0.8858 |
| **CUPRAC** | 0.10358 | 0.68309 | 0.7538 | 0.71285 | 0.72763 | 0.62052 | 0.74433 | 0.73284 | 0.13114 | 0.95986 | 0.9097 | 0.74388 | 0.07803 | 0.59202 | 0.65685 | 0.5205 | 0.00026178 | 0.94741 | 0.75572 | 0.43309 | 0.39095 | 0.44785 |
| **Neochlorogenic aci** | 0.024933 | 0.46489 | 0.49892 | 0.93933 | 0.9565 | 0.94929 | 0.76071 | 0.86482 | 0.12323 | 0.80407 | 0.98262 | 0.9206 | 0.072144 | 0.58131 | 0.97403 | 0.9599 | 0.01185 | 0.42695 | 0.57533 | 0.9354 | 0.77933 | 0.10004 |
| **Pelargonidin-3-glu** | 0.38138 | 0.9745 | 0.80175 | 392 | 0.42112 | 0.40313 | 0.98293 | 0.68131 | 0.15421 | 0.98036 | 0.4941 | 0.99356 | 0.20185 | 0.92488 | 0.41472 | 0.97357 | 0.00035433 | 0.96582 | 0.74533 | 0.51632 | 0.41219 | 0.72987 |
| **Glucosidase inhibi** | 0.46301 | 0.87396 | 0.96177 | 0.75265 | 0.77909 | 0.45751 | 0.9225 | 0.92418 | 0.44247 | 0.90492 | 0.027966 | 0.79974 | 0.57137 | 0.98213 | 0.86376 | 0.47282 | 0.29011 | 0.45867 | 0.59455 | 0.7138 | 0.073046 | 0.43918 |
| **TFC** | 0.87418 | 0.82554 | 0.67998 | 0.48374 | 0.51395 | 0.42132 | 0.0059218 | 0.16117 | 0.89468 | 0.46203 | 0.78553 | 0.47742 | 0.1595 | 0.43506 | 0.90048 | 0.52222 | 0.17431 | 0.74021 | 0.96482 | 0.97021 | 0.93509 | 0.89432 |
| **3,5-Dicaffeoylquin** | 0.5154 | 0.74583 | 0.76787 | 0.91831 | 0.89868 | 0.52368 | 0.85672 | 0.86426 | 0.30718 | 0.25184 | 0.79353 | 0.62366 | 0.89816 | 0.9065 | 0.37106 | 0.15313 | 0.7752 | 0.1388 | 0.17262 | 0.18651 | 0.65614 | 0.22681 |
| **Gallic acid** | 0.1172 | 0.40951 | 0.37365 | 0.60629 | 0.59932 | 0.74224 | 0.034595 | 0.24425 | 0.06756 | 0.78837 | 0.76617 | 0.19662 | 0.82163 | 0.50324 | 0.54285 | 0.20414 | 0.8088 | 0.054078 | 0.059065 | 0.099666 | 0.62229 | 0.031399 |
| **BChE inhibition** | 0.31429 | 0.68556 | 0.72149 | 0.99042 | 0.96871 | 0.13813 | 0.94569 | 0.95758 | 0.53022 | 0.78131 | 0.51235 | 0.7108 | 0.59516 | 0.99066 | 0.83721 | 0.26 | 0.70111 | 0.18199 | 0.24198 | 0.31194 | 0.16312 | 0.18573 |
| **Phloretin** | 0.0037451 | 0.15785 | 0.11782 | 0.34428 | 0.34 | 0.17518 | 0.8718 | 0.6556 | 0.4443 | 0.48134 | 0.57609 | 0.60205 | 0.3382 | 0.68179 | 0.18727 | 0.043642 | 0.58911 | 8,828E-02 | 0.0001116 | 0.0020111 | 0.11148 | 8.2838e-10 |
| **Vanillic acid** | 0.0013082 | 0.83132 | 0.63963 | 0.84326 | 0.86396 | 0.49457 | 0.86767 | 0.87432 | 0.32016 | 0.59902 | 0.46412 | 0.73266 | 0.78463 | 0.74235 | 0.18131 | 0.1858 | 0.16241 | 0.020807 | 0.011234 | 0.019597 | 0.1409 | 0.0087664 |
| **Ellagic acid** | 0.00049086 | 0.17048 | 0.13071 | 0.47545 | 0.47996 | 0.44458 | 0.61229 | 0.88649 | 0.21431 | 0.62616 | 0.49399 | 0.9204 | 0.073691 | 0.40173 | 0.42405 | 0.7896 | 0.29114 | 0.10765 | 0.11913 | 0.32365 | 0.66449 | 0.0020022 |
| **Hesperidin** | 0.0014983 | 0.19123 | 0.16378 | 0.52303 | 0.51496 | 0.40445 | 0.79612 | 0.80896 | 0.24193 | 0.56052 | 0.6073 | 0.77843 | 0.14351 | 0.57639 | 0.36892 | 0.21229 | 0.57767 | 0.0096314 | 0.015256 | 0.066324 | 0.31702 | 9.5518e-05 |
| **Isorhamnetin** | NA | 0.50369 | 0.36818 | 0.78051 | 0.80103 | 0.60959 | 0.62397 | 0.81941 | 0.97202 | 0.62358 | 0.40026 | 0.94367 | 0.18883 | 0.43661 | 0.30648 | 0.73719 | 0.76997 | 0.1004 | 0.08692 | 0.2206 | 0.49507 | 0.0035016 |
| **ABTS** | 0.50369 | NA | 5.7734e-10 | 5.1969e-07 | 3.0893e-07 | 0.22508 | 0.76894 | 0.52875 | 0.31995 | 0.75157 | 0.53084 | 0.97932 | 0.56915 | 0.6549 | 0.43749 | 0.80576 | 0.74593 | 0.31548 | 0.26826 | 0.36741 | 0.55491 | 0.13286 |
| **Chlorogenic acid** | 0.36818 | 5.7734e-10 | NA | 2.4526e-07 | 2.4446e-07 | 0.17099 | 0.70647 | 0.61214 | 0.3734 | 0.71901 | 0.41433 | 0.93659 | 0.54071 | 0.58211 | 0.33791 | 0.86904 | 0.61199 | 0.27926 | 0.21437 | 0.31316 | 0.4911 | 0.086258 |
| **DPPH** | 0.78051 | 5.1969e-07 | 2.4526e-07 | NA | 0 | 0.18576 | 0.7724 | 0.57743 | 0.56076 | 0.78702 | 0.45105 | 0.94526 | 0.84586 | 0.69576 | 0.37985 | 0.92384 | 0.34341 | 0.44366 | 0.3357 | 0.38653 | 0.50123 | 0.2555 |
| **TPC** | 0.80103 | 3.0893e-07 | 2.4446e-07 | 0 | NA | 0.19141 | 0.7884 | 0.56566 | 0.54512 | 0.78743 | 0.47515 | 0.96442 | 0.85164 | 0.7135 | 0.39267 | 0.88163 | 0.35467 | 0.4285 | 0.32855 | 0.37653 | 0.4987 | 0.25664 |

| | | | | | | | | | | | | | | | | | | | | | | | | | | | | | | | | | | | | | | | | | | | | |
|---|---|---|---|---|---|---|---|---|---|---|---|---|---|---|---|---|---|---|---|---|---|---|---|---|---|---|---|---|---|---|---|---|---|---|---|---|---|---|---|---|---|---|---|---|
| **Ferulic acid** | 0.78211 | 0.27222 | 0.14565 | 0.15282 | 0.67413 | 0.42684 | 0.74086 | 0.64138 | 0.29217 | 0.81869 | 0.62052 | 0.94929 | 0.40313 | 0.45751 | 0.42132 | 0.52368 | 0.74224 | 0.13813 | 0.17518 | 0.49457 | 0.44458 | 0.40445 | 0.60959 | 0.22508 | 0.17099 | 0.18576 | 0.19141 | NA | 0.87682 | 0.48351 | 0.47533 | 0.28717 | 0.26833 | 0.85615 | 0.90791 | 0.7955 | 0.11664 | 0.45576 | 0.19999 | 0.16889 | 0.12066 | 0.13656 | 0.051259 | 0.12493 |
| **FRAP** | 0.11563 | 0.54366 | 0.42443 | 0.59044 | 0.61206 | 0.70512 | 0.29936 | 0.47141 | 0.75845 | 0.89628 | 0.74433 | 0.76071 | 0.98293 | 0.9225 | 0.0059218 | 0.85672 | 0.034595 | 0.94569 | 0.8718 | 0.86767 | 0.61229 | 0.79612 | 0.62397 | 0.76894 | 0.70647 | 0.7724 | 0.7884 | 0.87682 | NA | 0.012597 | 0.18829 | 0.31645 | 0.6189 | 0.27599 | 0.37547 | 0.2904 | 0.4338 | 0.52597 | 0.82767 | 0.82025 | 0.88866 | 0.78937 | 0.87796 | 0.83419 |
| **Isoquercitrin** | 0.46595 | 0.093009 | 0.99495 | 0.50361 | 0.44985 | 0.75632 | 0.84991 | 0.70517 | 0.58587 | 0.77603 | 0.73284 | 0.86482 | 0.68131 | 0.92418 | 0.16117 | 0.86426 | 0.24425 | 0.95758 | 0.6556 | 0.87432 | 0.88649 | 0.80896 | 0.81941 | 0.52875 | 0.61214 | 0.57743 | 0.56566 | 0.48351 | 0.012597 | NA | 0.65142 | 0.3505 | 0.38078 | 0.3577 | 0.34879 | 0.26657 | 0.25787 | 0.68056 | 0.54515 | 0.5653 | 0.53814 | 0.52604 | 0.65116 | 0.60731 |
| **Epicatechin** | 0.21056 | 0.31684 | 0.4823 | 0.91304 | 0.99749 | 0.15043 | 0.77267 | 0.31339 | 0.20841 | 0.86215 | 0.13114 | 0.12323 | 0.15421 | 0.44247 | 0.89468 | 0.30718 | 0.06756 | 0.53022 | 0.4443 | 0.32016 | 0.21431 | 0.24193 | 0.97202 | 0.31995 | 0.3734 | 0.56076 | 0.54512 | 0.47533 | 0.18829 | 0.65142 | NA | 0.32826 | 0.67 | 0.6053 | 0.76618 | 0.73639 | 0.32812 | 0.99256 | 0.25507 | 0.77197 | 0.87045 | 0.96081 | 0.63589 | 0.54225 |
| **AChE inhibition** | 0.30005 | 0.77989 | 0.5281 | 0.52459 | 0.41733 | 0.64896 | 0.032652 | 0.90096 | 0.82627 | 0.54401 | 0.95986 | 0.80407 | 0.98036 | 0.90492 | 0.46203 | 0.25184 | 0.78837 | 0.78131 | 0.48134 | 0.59902 | 0.62616 | 0.56052 | 0.62358 | 0.75157 | 0.71901 | 0.78702 | 0.78743 | 0.28717 | 0.31645 | 0.3505 | 0.32826 | NA | 0.13295 | 0.050499 | 0.46522 | 0.37739 | 0.37976 | 0.64485 | 0.75418 | 0.50012 | 0.48826 | 0.52061 | 0.91999 | 0.47528 |
| **Metal chelating** | 0.70188 | 0.3215 | 0.26126 | 0.55805 | 0.62483 | 0.81249 | 0.3563 | 0.62986 | 0.75645 | 0.80997 | 0.9097 | 0.98262 | 0.4941 | 0.027966 | 0.78553 | 0.79353 | 0.76617 | 0.51235 | 0.57609 | 0.46412 | 0.49399 | 0.6073 | 0.40026 | 0.53084 | 0.41433 | 0.45105 | 0.47515 | 0.26833 | 0.6189 | 0.38078 | 0.67 | 0.13295 | NA | 0.024198 | 0.72633 | 0.71794 | 0.95137 | 0.62442 | 0.62929 | 0.83029 | 0.69197 | 0.78927 | 0.09188 | 0.46325 |
| **4-Hydroxy benzoic** | 0.26661 | 0.66074 | 0.37315 | 0.56858 | 0.39977 | 0.42026 | 0.27529 | 0.3272 | 0.56129 | 0.84675 | 0.74388 | 0.9206 | 0.99356 | 0.79974 | 0.47742 | 0.62366 | 0.19662 | 0.7108 | 0.60205 | 0.73266 | 0.9204 | 0.77843 | 0.94367 | 0.97932 | 0.93659 | 0.94526 | 0.96442 | 0.85615 | 0.27599 | 0.3577 | 0.6053 | 0.050499 | 0.024198 | NA | 0.40163 | 0.30751 | 0.27466 | 0.21364 | 0.56861 | 0.34453 | 0.38555 | 0.32992 | 0.54498 | 0.63968 |
| **Amylase inhibition** | 0.013205 | 0.29369 | 0.32404 | 0.28486 | 0.60826 | 0.80468 | 0.34239 | 0.098335 | 0.1501 | 0.32306 | 0.07803 | 0.072144 | 0.20185 | 0.57137 | 0.1595 | 0.89816 | 0.82163 | 0.59516 | 0.3382 | 0.78463 | 0.073691 | 0.14351 | 0.18883 | 0.56915 | 0.54071 | 0.84586 | 0.85164 | 0.90791 | 0.37547 | 0.34879 | 0.76618 | 0.46522 | 0.72633 | 0.40163 | NA | 2.0852e-05 | 0.049657 | 0.62192 | 0.22405 | 0.84886 | 0.88951 | 0.86014 | 0.85889 | 0.37649 |
| **Phosphomolybdenum** | 0.067053 | 0.40932 | 0.33102 | 0.48321 | 0.59074 | 0.72682 | 0.31554 | 0.36279 | 0.67299 | 0.81741 | 0.59202 | 0.58131 | 0.92488 | 0.98213 | 0.43506 | 0.9065 | 0.50324 | 0.99066 | 0.68179 | 0.74235 | 0.40173 | 0.57639 | 0.43661 | 0.6549 | 0.58211 | 0.69576 | 0.7135 | 0.7955 | 0.2904 | 0.26657 | 0.73639 | 0.37739 | 0.71794 | 0.30751 | 2.0852e-05 | NA | 0.012814 | 0.53339 | 0.77487 | 0.96521 | 0.9776 | 0.88475 | 0.93126 | 0.63141 |
| **Kaempferol-3-gluco** | 0.36408 | 0.43448 | 0.3714 | 0.56621 | 0.56297 | 0.96676 | 0.29918 | 0.70611 | 0.32378 | 0.98164 | 0.65685 | 0.97403 | 0.41472 | 0.86376 | 0.90048 | 0.37106 | 0.54285 | 0.83721 | 0.18727 | 0.18131 | 0.42405 | 0.36892 | 0.30648 | 0.43749 | 0.33791 | 0.37985 | 0.39267 | 0.11664 | 0.4338 | 0.25787 | 0.32812 | 0.37976 | 0.95137 | 0.27466 | 0.049657 | 0.012814 | NA | 0.51193 | 0.22442 | 0.18876 | 0.13679 | 0.15655 | 0.12109 | 0.13015 |
| **Pelargonidin-3-rut** | 0.2714 | 0.46799 | 0.034291 | 0.21727 | 0.017429 | 0.015471 | 0.1888 | 0.0085528 | 0.12253 | 0.65467 | 0.5205 | 0.9599 | 0.97357 | 0.47282 | 0.52222 | 0.15313 | 0.20414 | 0.26 | 0.043642 | 0.1858 | 0.7896 | 0.21229 | 0.73719 | 0.80576 | 0.86904 | 0.92384 | 0.88163 | 0.45576 | 0.52597 | 0.68056 | 0.99256 | 0.64485 | 0.62442 | 0.21364 | 0.62192 | 0.53339 | 0.51193 | NA | 0.1042 | 0.00022193 | 0.00099857 | 0.00016207 | 0.060103 | 0.060713 |
| **Procyanidin B2** | 0.1343 | 0.85658 | 0.54039 | 0.24807 | 0.32736 | 0.27548 | 0.43504 | 0.0016592 | 1.6631e-09 | 0.040551 | 0.00026178 | 0.01185 | 0.00035433 | 0.29011 | 0.17431 | 0.7752 | 0.8088 | 0.70111 | 0.58911 | 0.16241 | 0.29114 | 0.57767 | 0.76997 | 0.74593 | 0.61199 | 0.34341 | 0.35467 | 0.19999 | 0.82767 | 0.54515 | 0.25507 | 0.75418 | 0.62929 | 0.56861 | 0.22405 | 0.77487 | 0.22442 | 0.1042 | NA | 0.11503 | 0.05561 | 0.017712 | 0.073094 | 0.38979 |
| **Malvidin-3-galacto** | 0.99583 | 0.68138 | 0.44203 | 0.71202 | 0.081869 | 0.029669 | 0.22617 | 0.16469 | 0.21418 | 0.79468 | 0.94741 | 0.42695 | 0.96582 | 0.45867 | 0.74021 | 0.1388 | 0.054078 | 0.18199 | 8,828E-02 | 0.020807 | 0.10765 | 0.0096314 | 0.1004 | 0.31548 | 0.27926 | 0.44366 | 0.4285 | 0.16889 | 0.82025 | 0.5653 | 0.77197 | 0.50012 | 0.83029 | 0.34453 | 0.84886 | 0.96521 | 0.18876 | 0.00022193 | 0.11503 | NA | 9.1674e-10 | 1.0769e-07 | 0.029542 | 0.00010947 |
| **Delphindin-3-galac** | 0.97598 | 0.56984 | 0.59321 | 0.75164 | 0.11348 | 0.040169 | 0.23712 | 0.15702 | 0.12682 | 0.64773 | 0.75572 | 0.57533 | 0.74533 | 0.59455 | 0.96482 | 0.17262 | 0.059065 | 0.24198 | 0.0001116 | 0.011234 | 0.11913 | 0.015256 | 0.08692 | 0.26826 | 0.21437 | 0.3357 | 0.32855 | 0.12066 | 0.88866 | 0.53814 | 0.87045 | 0.48826 | 0.69197 | 0.38555 | 0.88951 | 0.9776 | 0.13679 | 0.00099857 | 0.05561 | 9.1674e-10 | NA | 7.5817e-09 | 0.023082 | 5,467E-02 |
| **Tyrosinase inhibit** | 0.67789 | 0.80837 | 0.37831 | 0.51504 | 0.074317 | 0.029288 | 0.21191 | 0.044332 | 0.043855 | 0.47416 | 0.43309 | 0.9354 | 0.51632 | 0.7138 | 0.97021 | 0.18651 | 0.099666 | 0.31194 | 0.0020111 | 0.019597 | 0.32365 | 0.066324 | 0.2206 | 0.36741 | 0.31316 | 0.38653 | 0.37653 | 0.13656 | 0.78937 | 0.52604 | 0.96081 | 0.52061 | 0.78927 | 0.32992 | 0.86014 | 0.88475 | 0.15655 | 0.00016207 | 0.017712 | 1.0769e-07 | 7.5817e-09 | NA | 0.017288 | 0.0012533 |
| **Hyperoside** | 0.69593 | 0.86478 | 0.79636 | 0.80558 | 0.58205 | 0.36633 | 0.77722 | 0.15169 | 0.10918 | 0.92046 | 0.39095 | 0.77933 | 0.41219 | 0.073046 | 0.93509 | 0.65614 | 0.62229 | 0.16312 | 0.11148 | 0.1409 | 0.66449 | 0.31702 | 0.49507 | 0.55491 | 0.4911 | 0.50123 | 0.4987 | 0.051259 | 0.87796 | 0.65116 | 0.63589 | 0.91999 | 0.09188 | 0.54498 | 0.85889 | 0.93126 | 0.12109 | 0.060103 | 0.073094 | 0.029542 | 0.023082 | 0.017288 | NA | 0.087288 |
| **Delphindin-3,5- di** | 0.25373 | 0.16333 | 0.6641 | 0.58782 | 0.39479 | 0.15771 | 0.40435 | 0.97269 | 0.67371 | 0.8858 | 0.44785 | 0.10004 | 0.72987 | 0.43918 | 0.89432 | 0.22681 | 0.031399 | 0.18573 | 8.2838e-10 | 0.0087664 | 0.0020022 | 9.5518e-05 | 0.0035016 | 0.13286 | 0.086258 | 0.2555 | 0.25664 | 0.12493 | 0.83419 | 0.60731 | 0.54225 | 0.47528 | 0.46325 | 0.63968 | 0.37649 | 0.63141 | 0.13015 | 0.060713 | 0.38979 | 0.00010947 | 5,467E-02 | 0.0012533 | 0.087288 | NA |

# **Table S6.** *P*-value between bioactive compounds and enzyme inhibition assays.

| | Ferulic acid | Chlorogenic acid | Phloretin | Vanillic acid | 3,5-Dicaffeoylquin | Gallic acid | BChE inhibition | 4-Hydroxy benzoic | Pelargonidin-3-glu | Neochlorogenic aci | Glucosidase inhibi | Ellagic acid | Hesperidin | Isorhamnetin | Pelargonidin-3-rut | Procyanidin B2 | Isoquercitrin | Epicatechin |
|---|---|---|---|---|---|---|---|---|---|---|---|---|---|---|---|---|---|---|
| **Ferulic acid** | NA | 0.17099 | 0.17518 | 0.49457 | 0.52368 | 0.74224 | 0.13813 | 0.85615 | 0.40313 | 0.94929 | 0.45751 | 0.44458 | 0.40445 | 0.60959 | 0.45576 | 0.19999 | 0.48351 | 0.47533 |
| **Chlorogenic acid** | 0.17099 | NA | 0.11782 | 0.63963 | 0.76787 | 0.37365 | 0.72149 | 0.93659 | 0.80175 | 0.49892 | 0.96177 | 0.13071 | 0.16378 | 0.36818 | 0.86904 | 0.61199 | 0.61214 | 0.3734 |
| **Phloretin** | 0.17518 | 0.11782 | NA | 0.016137 | 0.18786 | 0.025434 | 0.13365 | 0.60205 | 0.45221 | 0.045483 | 0.31346 | 0.00097963 | 8.94e-06 | 0.0037451 | 0.043642 | 0.58911 | 0.6556 | 0.4443 |
| **Vanillic acid** | 0.49457 | 0.63963 | 0.016137 | NA | 0.45923 | 0.18536 | 0.51438 | 0.73266 | 0.57249 | 0.66423 | 0.89177 | 0.14151 | 0.079321 | 0.0013082 | 0.1858 | 0.16241 | 0.87432 | 0.32016 |
| **3,5-Dicaffeoylquin** | 0.52368 | 0.76787 | 0.18786 | 0.45923 | NA | 0.030704 | 0.81622 | 0.62366 | 0.60691 | 0.49878 | 0.92794 | 0.44566 | 0.20452 | 0.5154 | 0.15313 | 0.7752 | 0.86426 | 0.30718 |
| **Gallic acid** | 0.74224 | 0.37365 | 0.025434 | 0.18536 | 0.030704 | NA | 0.31906 | 0.19662 | 0.5361 | 0.19867 | 0.4736 | 0.079631 | 0.038212 | 0.1172 | 0.20414 | 0.8088 | 0.24425 | 0.06756 |
| **BChE inhibition** | 0.13813 | 0.72149 | 0.13365 | 0.51438 | 0.81622 | 0.31906 | NA | 0.7108 | 0.22874 | 0.15919 | 0.90088 | 0.1872 | 0.13481 | 0.31429 | 0.26 | 0.70111 | 0.95758 | 0.53022 |
| **4-Hydroxy benzoic** | 0.85615 | 0.93659 | 0.60205 | 0.73266 | 0.62366 | 0.19662 | 0.7108 | NA | 0.99356 | 0.9206 | 0.79974 | 0.9204 | 0.77843 | 0.94367 | 0.21364 | 0.56861 | 0.3577 | 0.6053 |
| **Pelargonidin-3-glu** | 0.40313 | 0.80175 | 0.45221 | 0.57249 | 0.60691 | 0.5361 | 0.22874 | 0.99356 | NA | 0.00017634 | 0.076278 | 0.068555 | 0.076892 | 0.38138 | 0.97357 | 0.00035433 | 0.68131 | 0.15421 |
| **Neochlorogenic aci** | 0.94929 | 0.49892 | 0.045483 | 0.66423 | 0.49878 | 0.19867 | 0.15919 | 0.9206 | 0.00017634 | NA | 0.094697 | 0.0001767 | 0.00088173 | 0.024933 | 0.9599 | 0.01185 | 0.86482 | 0.12323 |
| **Glucosidase inhibi** | 0.45751 | 0.96177 | 0.31346 | 0.89177 | 0.92794 | 0.4736 | 0.90088 | 0.79974 | 0.076278 | 0.094697 | NA | 0.23722 | 0.20905 | 0.46301 | 0.47282 | 0.29011 | 0.92418 | 0.44247 |
| **Ellagic acid** | 0.44458 | 0.13071 | 0.00097963 | 0.14151 | 0.44566 | 0.079631 | 0.1872 | 0.9204 | 0.068555 | 0.0001767 | 0.23722 | NA | 2.8649e-06 | 0.00049086 | 0.7896 | 0.29114 | 0.88649 | 0.21431 |
| **Hesperidin** | 0.40445 | 0.16378 | 8.94e-06 | 0.079321 | 0.20452 | 0.038212 | 0.13481 | 0.77843 | 0.076892 | 0.00088173 | 0.20905 | 2.8649e-06 | NA | 0.0014983 | 0.21229 | 0.57767 | 0.80896 | 0.24193 |
| **Isorhamnetin** | 0.60959 | 0.36818 | 0.0037451 | 0.0013082 | 0.5154 | 0.1172 | 0.31429 | 0.94367 | 0.38138 | 0.024933 | 0.46301 | 0.00049086 | 0.0014983 | NA | 0.73719 | 0.76997 | 0.81941 | 0.97202 |
| **Pelargonidin-3-rut** | 0.45576 | 0.86904 | 0.043642 | 0.1858 | 0.15313 | 0.20414 | 0.26 | 0.21364 | 0.97357 | 0.9599 | 0.47282 | 0.7896 | 0.21229 | 0.73719 | NA | 0.1042 | 0.68056 | 0.99256 |
| **Procyanidin B2** | 0.19999 | 0.61199 | 0.58911 | 0.16241 | 0.7752 | 0.8088 | 0.70111 | 0.56861 | 0.00035433 | 0.01185 | 0.29011 | 0.29114 | 0.57767 | 0.76997 | 0.1042 | NA | 0.54515 | 0.25507 |
| **Isoquercitrin** | 0.48351 | 0.61214 | 0.6556 | 0.87432 | 0.86426 | 0.24425 | 0.95758 | 0.3577 | 0.68131 | 0.86482 | 0.92418 | 0.88649 | 0.80896 | 0.81941 | 0.68056 | 0.54515 | NA | 0.65142 |
| **Epicatechin** | 0.47533 | 0.3734 | 0.4443 | 0.32016 | 0.30718 | 0.06756 | 0.53022 | 0.6053 | 0.15421 | 0.12323 | 0.44247 | 0.21431 | 0.24193 | 0.97202 | 0.99256 | 0.25507 | 0.65142 | NA |
| **AChE inhibition** | 0.28717 | 0.71901 | 0.48134 | 0.59902 | 0.25184 | 0.78837 | 0.78131 | 0.050499 | 0.98036 | 0.80407 | 0.90492 | 0.62616 | 0.56052 | 0.62358 | 0.64485 | 0.75418 | 0.3505 | 0.32826 |
| **Caffeic acid** | 0.74086 | 0.41659 | 0.39271 | 0.23459 | 0.043498 | 0.42576 | 0.6599 | 0.27529 | 0.89005 | 0.97701 | 0.82385 | 0.86731 | 0.59157 | 0.54004 | 0.1888 | 0.43504 | 0.84991 | 0.77267 |
| **Amylase inhibition** | 0.90791 | 0.54071 | 0.3382 | 0.78463 | 0.89816 | 0.82163 | 0.59516 | 0.40163 | 0.20185 | 0.072144 | 0.57137 | 0.073691 | 0.14351 | 0.18883 | 0.62192 | 0.22405 | 0.34879 | 0.76618 |
| **Kaempferol-3-gluco** | 0.11664 | 0.33791 | 0.18727 | 0.18131 | 0.37106 | 0.54285 | 0.83721 | 0.27466 | 0.41472 | 0.97403 | 0.86376 | 0.42405 | 0.36892 | 0.30648 | 0.51193 | 0.22442 | 0.25787 | 0.32812 |
| **Malvidin-3-galacto** | 0.16889 | 0.27926 | 8,828E-02 | 0.020807 | 0.1388 | 0.054078 | 0.18199 | 0.34453 | 0.96582 | 0.42695 | 0.45867 | 0.10765 | 0.0096314 | 0.1004 | 0.00022193 | 0.11503 | 0.5653 | 0.77197 |
| **Delphinidin-3-galac** | 0.12066 | 0.21437 | 0.0001116 | 0.011234 | 0.17262 | 0.059065 | 0.24198 | 0.38555 | 0.74533 | 0.57533 | 0.59455 | 0.11913 | 0.015256 | 0.08692 | 0.00099857 | 0.05561 | 0.53814 | 0.87045 |
| **Tyrosinase inhibit** | 0.13656 | 0.31316 | 0.0020111 | 0.019597 | 0.18651 | 0.099666 | 0.31194 | 0.32992 | 0.51632 | 0.9354 | 0.7138 | 0.32365 | 0.066324 | 0.2206 | 0.00016207 | 0.017712 | 0.52604 | 0.96081 |
| **Hyperoside** | 0.051259 | 0.4911 | 0.11148 | 0.1409 | 0.65614 | 0.62229 | 0.16312 | 0.54498 | 0.41219 | 0.77933 | 0.073046 | 0.66449 | 0.31702 | 0.49507 | 0.060103 | 0.073094 | 0.65116 | 0.63589 |
| **Delphinidin-3,5- di** | 0.12493 | 0.086258 | 8.2838e-10 | 0.0087664 | 0.22681 | 0.031399 | 0.18573 | 0.63968 | 0.72987 | 0.10004 | 0.43918 | 0.0020022 | 9.5518e-05 | 0.0035016 | 0.060713 | 0.38979 | 0.60731 | 0.54225 |
| **Quercetin** | 0.64138 | 0.63523 | 0.95734 | 0.63164 | 0.60615 | 0.97182 | 0.89445 | 0.3272 | 0.056098 | 0.014897 | 0.6035 | 0.04826 | 0.30998 | 0.1876 | 0.0085528 | 0.0016592 | 0.70517 | 0.31339 |
| **Syringic acid** | 0.29217 | 0.82006 | 0.89953 | 0.2915 | 0.85629 | 0.99163 | 0.59752 | 0.56129 | 0.00018443 | 0.0025125 | 0.25123 | 0.12358 | 0.32718 | 0.48051 | 0.12253 | 1.6631e-09 | 0.58587 | 0.20841 |
| **Quercitrin** | 0.81869 | 0.96622 | 0.71718 | 0.69123 | 0.9297 | 0.91044 | 0.95832 | 0.84675 | 0.03294 | 0.050509 | 0.77254 | 0.21719 | 0.24301 | 0.49857 | 0.65467 | 0.040551 | 0.77603 | 0.86215 |
| **Phloridzin** | 0.67413 | 0.14439 | 0.33948 | 0.24747 | 0.015216 | 0.14529 | 0.50179 | 0.39977 | 0.96564 | 0.89806 | 0.64784 | 0.89864 | 0.59639 | 0.76471 | 0.017429 | 0.32736 | 0.44985 | 0.99749 |
| **Catechin** | 0.42684 | 0.8074 | 0.1382 | 0.30929 | 0.044283 | 0.12583 | 0.42619 | 0.42026 | 0.98628 | 0.89297 | 0.61903 | 0.7173 | 0.31753 | 0.68543 | 0.015471 | 0.27548 | 0.75632 | 0.15043 |
| **Kaempferol** | 0.78211 | 0.31746 | 0.24047 | 0.61743 | 0.98667 | 0.19566 | 0.61716 | 0.26661 | 0.18738 | 0.02205 | 0.56461 | 0.010212 | 0.067284 | 0.046878 | 0.2714 | 0.1343 | 0.46595 | 0.21056 |
| **p-Coumaric acid** | 0.27222 | 0.73239 | 0.21008 | 0.77929 | 0.99629 | 0.48189 | 0.83999 | 0.66074 | 0.95227 | 0.28263 | 0.97492 | 0.060757 | 0.16362 | 0.24075 | 0.46799 | 0.85658 | 0.093009 | 0.31684 |
| **Rutin** | 0.14565 | 0.30453 | 0.79739 | 0.70547 | 0.20493 | 0.76148 | 0.73599 | 0.37315 | 0.87138 | 0.46831 | 0.71252 | 0.18055 | 0.57329 | 0.18964 | 0.034291 | 0.54039 | 0.99495 | 0.4823 |
| **Cyanidin-3-glucosi** | 0.15282 | 0.016603 | 0.59398 | 0.7153 | 0.40375 | 0.95313 | 0.87275 | 0.56858 | 0.42998 | 0.16503 | 0.80379 | 0.1219 | 0.31927 | 0.4274 | 0.21727 | 0.24807 | 0.50361 | 0.91304 |

| | AChE inhibition | Caffeic acid | Amylase inhibition | Kaempferol-3-gluco | Malvidin-3-galacto | Delphinidin-3-galac | Tyrosinase inhibit | Hyperoside | Delphinidin-3,5- di | Quercetin | Syringic acid | Quercitrin | Phloridzin | Catechin | Kaempferol | p-Coumaric acid | Rutin | Cyanidin-3-glucosi |
|---|---|---|---|---|---|---|---|---|---|---|---|---|---|---|---|---|---|---|
| **Ferulic acid** | 0.28717 | 0.74086 | 0.90791 | 0.11664 | 0.16889 | 0.12066 | 0.13656 | 0.051259 | 0.12493 | 0.64138 | 0.29217 | 0.81869 | 0.67413 | 0.42684 | 0.78211 | 0.27222 | 0.14565 | 0.15282 |
| **Chlorogenic acid** | 0.71901 | 0.41659 | 0.54071 | 0.33791 | 0.27926 | 0.21437 | 0.31316 | 0.4911 | 0.086258 | 0.63523 | 0.82006 | 0.96622 | 0.14439 | 0.8074 | 0.31746 | 0.73239 | 0.30453 | 0.016603 |
| **Phloretin** | 0.48134 | 0.39271 | 0.3382 | 0.18727 | 8,828E-02 | 0.0001116 | 0.0020111 | 0.11148 | 8.2838e-10 | 0.95734 | 0.89953 | 0.71718 | 0.33948 | 0.1382 | 0.24047 | 0.21008 | 0.79739 | 0.59398 |
| **Vanillic acid** | 0.59902 | 0.23459 | 0.78463 | 0.18131 | 0.020807 | 0.011234 | 0.019597 | 0.1409 | 0.0087664 | 0.63164 | 0.2915 | 0.69123 | 0.24747 | 0.30929 | 0.61743 | 0.77929 | 0.70547 | 0.7153 |
| **3,5-Dicaffeoylquin** | 0.25184 | 0.043498 | 0.89816 | 0.37106 | 0.1388 | 0.17262 | 0.18651 | 0.65614 | 0.22681 | 0.60615 | 0.85629 | 0.9297 | 0.015216 | 0.044283 | 0.98667 | 0.99629 | 0.20493 | 0.40375 |
| **Gallic acid** | 0.78837 | 0.42576 | 0.82163 | 0.54285 | 0.054078 | 0.059065 | 0.099666 | 0.62229 | 0.031399 | 0.97182 | 0.99163 | 0.91044 | 0.14529 | 0.12583 | 0.19566 | 0.48189 | 0.76148 | 0.95313 |
| **BChE inhibition** | 0.78131 | 0.6599 | 0.59516 | 0.83721 | 0.18199 | 0.24198 | 0.31194 | 0.16312 | 0.18573 | 0.89445 | 0.59752 | 0.95832 | 0.50179 | 0.42619 | 0.61716 | 0.83999 | 0.73599 | 0.87275 |
| **4-Hydroxy benzoic** | 0.050499 | 0.27529 | 0.40163 | 0.27466 | 0.34453 | 0.38555 | 0.32992 | 0.54498 | 0.63968 | 0.3272 | 0.56129 | 0.84675 | 0.39977 | 0.42026 | 0.26661 | 0.66074 | 0.37315 | 0.56858 |
| **Pelargonidin-3-glu** | 0.98036 | 0.89005 | 0.20185 | 0.41472 | 0.96582 | 0.74533 | 0.51632 | 0.41219 | 0.72987 | 0.056098 | 0.00018443 | 0.03294 | 0.96564 | 0.98628 | 0.18738 | 0.95227 | 0.87138 | 0.42998 |
| **Neochlorogenic aci** | 0.80407 | 0.97701 | 0.072144 | 0.97403 | 0.42695 | 0.57533 | 0.9354 | 0.77933 | 0.10004 | 0.014897 | 0.0025125 | 0.050509 | 0.89806 | 0.89297 | 0.02205 | 0.28263 | 0.46831 | 0.16503 |
| **Glucosidase inhibi** | 0.90492 | 0.82385 | 0.57137 | 0.86376 | 0.45867 | 0.59455 | 0.7138 | 0.073046 | 0.43918 | 0.6035 | 0.25123 | 0.77254 | 0.64784 | 0.61903 | 0.56461 | 0.97492 | 0.71252 | 0.80379 |
| **Ellagic acid** | 0.62616 | 0.86731 | 0.073691 | 0.42405 | 0.10765 | 0.11913 | 0.32365 | 0.66449 | 0.0020022 | 0.04826 | 0.12358 | 0.21719 | 0.89864 | 0.7173 | 0.010212 | 0.060757 | 0.18055 | 0.1219 |
| **Hesperidin** | 0.56052 | 0.59157 | 0.14351 | 0.36892 | 0.0096314 | 0.015256 | 0.066324 | 0.31702 | 9.5518e-05 | 0.30998 | 0.32718 | 0.24301 | 0.59639 | 0.31753 | 0.067284 | 0.16362 | 0.57329 | 0.31927 |
| **Isorhamnetin** | 0.62358 | 0.54004 | 0.18883 | 0.30648 | 0.1004 | 0.08692 | 0.2206 | 0.49507 | 0.0035016 | 0.1876 | 0.48051 | 0.49857 | 0.76471 | 0.68543 | 0.046878 | 0.24075 | 0.18964 | 0.4274 |
| **Pelargonidin-3-rut** | 0.64485 | 0.1888 | 0.62192 | 0.51193 | 0.00022193 | 0.00099857 | 0.00016207 | 0.060103 | 0.060713 | 0.0085528 | 0.12253 | 0.65467 | 0.017429 | 0.015471 | 0.2714 | 0.46799 | 0.034291 | 0.21727 |
| **Procyanidin B2** | 0.75418 | 0.43504 | 0.22405 | 0.22442 | 0.11503 | 0.05561 | 0.017712 | 0.073094 | 0.38979 | 0.0016592 | 1.6631e-09 | 0.040551 | 0.32736 | 0.27548 | 0.1343 | 0.85658 | 0.54039 | 0.24807 |
| **Isoquercitrin** | 0.3505 | 0.84991 | 0.34879 | 0.25787 | 0.5653 | 0.53814 | 0.52604 | 0.65116 | 0.60731 | 0.70517 | 0.58587 | 0.77603 | 0.44985 | 0.75632 | 0.46595 | 0.093009 | 0.99495 | 0.50361 |
| **Epicatechin** | 0.32826 | 0.77267 | 0.76618 | 0.32812 | 0.77197 | 0.87045 | 0.96081 | 0.63589 | 0.54225 | 0.31339 | 0.20841 | 0.86215 | 0.99749 | 0.15043 | 0.21056 | 0.31684 | 0.4823 | 0.91304 |
| **AChE inhibition** | NA | 0.032652 | 0.46522 | 0.37976 | 0.50012 | 0.48826 | 0.52061 | 0.91999 | 0.47528 | 0.90096 | 0.82627 | 0.54401 | 0.41733 | 0.64896 | 0.30005 | 0.77989 | 0.5281 | 0.52459 |
| **Caffeic acid** | 0.032652 | NA | 0.34239 | 0.29918 | 0.22617 | 0.23712 | 0.21191 | 0.77722 | 0.40435 | 0.34809 | 0.46712 | 0.44982 | 0.18161 | 0.11914 | 0.14877 | 0.18579 | 0.25976 | 0.66217 |
| **Amylase inhibition** | 0.46522 | 0.34239 | NA | 0.049657 | 0.84886 | 0.88951 | 0.86014 | 0.85889 | 0.37649 | 0.098335 | 0.1501 | 0.32306 | 0.60826 | 0.80468 | 0.013205 | 0.29369 | 0.32404 | 0.28486 |
| **Kaempferol-3-gluco** | 0.37976 | 0.29918 | 0.049657 | NA | 0.18876 | 0.13679 | 0.15655 | 0.12109 | 0.13015 | 0.70611 | 0.32378 | 0.98164 | 0.56297 | 0.96676 | 0.36408 | 0.43448 | 0.3714 | 0.56621 |
| **Malvidin-3-galacto** | 0.50012 | 0.22617 | 0.84886 | 0.18876 | NA | 9.1674e-10 | 1.0769e-07 | 0.029542 | 0.00010947 | 0.16469 | 0.21418 | 0.79468 | 0.081869 | 0.029669 | 0.99583 | 0.68138 | 0.44203 | 0.71202 |
| **Delphinidin-3-galac** | 0.48826 | 0.23712 | 0.88951 | 0.13679 | 9.1674e-10 | NA | 7.5817e-09 | 0.023082 | 5,467E-02 | 0.15702 | 0.12682 | 0.64773 | 0.11348 | 0.040169 | 0.97598 | 0.56984 | 0.59321 | 0.75164 |
| **Tyrosinase inhibit** | 0.52061 | 0.21191 | 0.86014 | 0.15655 | 1.0769e-07 | 7.5817e-09 | NA | 0.017288 | 0.0012533 | 0.044332 | 0.043855 | 0.47416 | 0.074317 | 0.029288 | 0.67789 | 0.80837 | 0.37831 | 0.51504 |
| **Hyperoside** | 0.91999 | 0.77722 | 0.85889 | 0.12109 | 0.029542 | 0.023082 | 0.017288 | NA | 0.087288 | 0.15169 | 0.10918 | 0.92046 | 0.58205 | 0.36633 | 0.69593 | 0.86478 | 0.79636 | 0.80558 |
| **Delphinidin-3,5- di** | 0.47528 | 0.40435 | 0.37649 | 0.13015 | 0.00010947 | 5,467E-02 | 0.0012533 | 0.087288 | NA | 0.97269 | 0.67371 | 0.8858 | 0.39479 | 0.15771 | 0.25373 | 0.16333 | 0.6641 | 0.58782 |
| **Quercetin** | 0.90096 | 0.34809 | 0.098335 | 0.70611 | 0.16469 | 0.15702 | 0.044332 | 0.15169 | 0.97269 | NA | 0.00036184 | 0.1088 | 0.080186 | 0.13311 | 0.0073489 | 0.14671 | 0.022971 | 0.04082 |
| **Syringic acid** | 0.82627 | 0.46712 | 0.1501 | 0.32378 | 0.21418 | 0.12682 | 0.043855 | 0.10918 | 0.67371 | 0.00036184 | NA | 0.033087 | 0.32125 | 0.31442 | 0.063848 | 0.63091 | 0.39431 | 0.16988 |
| **Quercitrin** | 0.54401 | 0.44982 | 0.32306 | 0.98164 | 0.79468 | 0.64773 | 0.47416 | 0.92046 | 0.8858 | 0.1088 | 0.033087 | NA | 0.42645 | 0.082995 | 0.24756 | 0.76377 | 0.18536 | 0.063797 |
| **Phloridzin** | 0.41733 | 0.18161 | 0.60826 | 0.56297 | 0.081869 | 0.11348 | 0.074317 | 0.58205 | 0.39479 | 0.080186 | 0.32125 | 0.42645 | NA | 0.026525 | 0.32963 | 0.86688 | 0.032112 | 0.0061434 |
| **Catechin** | 0.64896 | 0.11914 | 0.80468 | 0.96676 | 0.029669 | 0.040169 | 0.029288 | 0.36633 | 0.15771 | 0.13311 | 0.31442 | 0.082995 | 0.026525 | NA | 0.52103 | 0.73757 | 0.0042673 | 0.034514 |
| **Kaempferol** | 0.30005 | 0.14877 | 0.013205 | 0.36408 | 0.99583 | 0.97598 | 0.67789 | 0.69593 | 0.25373 | 0.0073489 | 0.063848 | 0.24756 | 0.32963 | 0.52103 | NA | 0.096234 | 0.056063 | 0.085045 |
| **p-Coumaric acid** | 0.77989 | 0.18579 | 0.29369 | 0.43448 | 0.68138 | 0.56984 | 0.80837 | 0.86478 | 0.16333 | 0.14671 | 0.63091 | 0.76377 | 0.86688 | 0.73757 | 0.096234 | NA | 0.072532 | 0.55562 |
| **Rutin** | 0.5281 | 0.25976 | 0.32404 | 0.3714 | 0.44203 | 0.59321 | 0.37831 | 0.79636 | 0.6641 | 0.022971 | 0.39431 | 0.18536 | 0.032112 | 0.0042673 | 0.056063 | 0.072532 | NA | 0.0044745 |
| **Cyanidin-3-glucosi** | 0.52459 | 0.66217 | 0.28486 | 0.56621 | 0.71202 | 0.75164 | 0.51504 | 0.80558 | 0.58782 | 0.04082 | 0.16988 | 0.063797 | 0.0061434 | 0.034514 | 0.085045 | 0.55562 | 0.0044745 | NA |
